# Ultra‐Thin and Highly Insulating Aromatic Monolayers by *N*‐Heterocyclic Carbenes

**DOI:** 10.1002/anie.4614418

**Published:** 2026-06-10

**Authors:** Mateusz Wróbel, Raka Ahmed, William Bro‐Jørgensen, Krzysztof Kozieł, Christian A. Nijhuis, Gemma C. Solomon, Piotr Cyganik

**Affiliations:** ^1^ Faculty of Physics, Astronomy and Applied Computer Science, Smoluchowski Institute of Physics Jagiellonian University Krakow Poland; ^2^ Department of Chemistry and Nano‐Science Center University of Copenhagen Copenhagen Denmark; ^3^ Faculty of Chemistry Jagiellonian University Krakow Poland; ^4^ Department of Molecules and Materials MESA+ Institute for Nanotechnology Molecules Center and Center for Brain‐Inspired Nano Systems Faculty of Science and Technology University of Twente Enschede AE the Netherlands; ^5^ NNF Quantum Computing Programme, Niels Bohr Institute University of Copenhagen Copenhagen Denmark

**Keywords:** conductivity, EGaIn, molecular insulator, molecular junction, *N*‐heterocyclic carbenes, self‐assembled monolayers

## Abstract

The efficiency of organic electronic devices relies on application of organic gate dielectric materials. Such organic films should exhibit high chemical/thermal stability, aromatic functionality compatible with organic semiconductors, and low gate leakage currents in combination with low thickness to reduce the operating voltage. An interesting class of materials for such applications are self‐assembled monolayers (SAMs) among which the *N*‐heterocyclic carbenes (NHC) are known for their high chemical/thermal stability. The conductivity of NHC SAMs, however, has been sparsely explored and their electrical properties remain controversial. Here we report conductivity analysis for a well‐defined series of aromatic NHC SAMs. Our data show that all analyzed monolayers are highly insulating and in particular the shortest possible NHC of just ∼3.3 Å is by 5 orders of magnitude more insulating than standard insulators based on alkanethiolate SAM of the same length. Our calculations indicate the absence of destructive quantum interference (DQI) effect which has been considered responsible for suppression of conductivity in aromatic molecules. The suppression of SAMs conductivity just via selection of the imidazolium‐based bonding group is conceptually simpler opening possibility of using NHC SAMs as an ultra‐thin, and exceptionally insulating, aromatic monolayers for functionalization of the gate electrodes.

## Graphical abstract

By using N‐heterocyclic carbenes extremally insulating and at the same time as thin as 0.3 nm molecular films are formed. The charge transport calculations indicate absence of destructive quantum interference effect which was so far the only way to suppress conductivity in aromatic molecules. Thus, a new possibility for functionalization of the gate electrodes in organic electronic devices is opened.

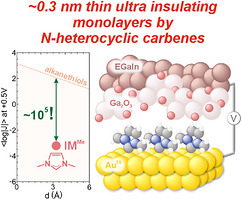

## 1 Introduction

Ultrathin organic gate dielectric materials represent a key strategy for enhancing the performance of organic electronic devices such as organic field effect transistor (OFET) [1, 2, 3]. Reducing the dielectric thickness improves device performance because it reduces operating bias voltage. This approach, however, is complicated by unwanted quantum mechanical tunneling resulting in leakage currents across the gate dielectric compromising performance. Therefore, the formation of highly insulating, ultrathin aromatic films on metal electrodes is essential to optimize this trade‐off between leakage across the gate dielectric and low operating voltage. Although polymer‐based dielectrics have been extensively studied and widely applied in OFETs [4], the fabrication of insulating films with thicknesses below 1 nm requires fundamentally different approach. Here we show that this can be effectively achieved through the use of self‐assembled monolayers (SAMs) with carbene anchoring groups. Our results highlight the potential role of carbene chemistry to control charge transport rates across electrode—molecule interfaces which may be important for future studies involving interfacial charge transport [1, 2, 3]. SAMs are a form of organic nanostructures that find applications ranging from molecular/organic electronic devices [5, 6, 7, 8, 9, 10, 11, 12, 13, 14] to design of new sensors and in tissue engineering [15, 16, 17, 18]. The design of new types of SAMs for electronics applications has mainly focused on improving functionality or conductivity, but in various applications, it is crucial to find alternatives and to combat current leakage via tunneling in nanoelectronics [3, 19, 20, 21, 22]. Of equal importance is to improve the thermal, chemical, or electrochemical stability of molecular structures in electronic devices [5, 7, 10, 14]. In this context, *N*‐heterocyclic carbenes (NHC) based SAMs [23, 24, 25, 26, 27, 28] that readily form on metals [25, 29, 30], semiconductors [31] or metal‐oxides [32, 33] are highly promising due to their exceptionally high chemical [29], electrochemical [29, 34, 35], and thermal [34, 36, 37, 38] stability. These features make NHC SAMs a very appealing alternative to, for example, thiols, for a range of applications including biological sensors [34, 39, 40, 41, 42], corrosion inhibition or surface passivation [33, 43, 44], surface patterning/modification [45, 46, 47, 48], and heterogeneous catalysis [25, 32, 49]. The potential applications of NHC SAMs in electronic devices remain, however, sparsely explored with contradictory conclusions claiming NHC junctions are highly conductive or insulating [37, 50, 51, 52]. Here, we demonstrate that the shortest possible NHC, with a length of only ∼3.3 Å, is more insulating by five orders of magnitude than an alkanethiolate SAMs of comparable length. As discussed in this work, enhanced insulating behavior in aromatic systems has previously been achieved—albeit to a much lesser extent—through destructive quantum interference (DQI), typically using significantly longer molecules. In contrast, our data indicate that DQI does not play a role in the present case. Instead, we propose an alternative approach based on a tailored imidazolium anchoring group. Such highly insulating small molecules are interesting for applications as ultrathin organic dielectrics where it is important to suppress unwanted tunneling, especially challenging in nanometer‐scale electronics. This strategy demonstrates clear novelty as it enables a reduction in organic dielectric thickness by a factor of 2–3 while simultaneously achieving record‐high resistivity in combination with the exceptionally high stability for NHC monolayers. Our findings are interesting for future studies involving charge transport across molecule—electrode interfaces where it is important to block unwanted leakage.

**FIGURE 1:**
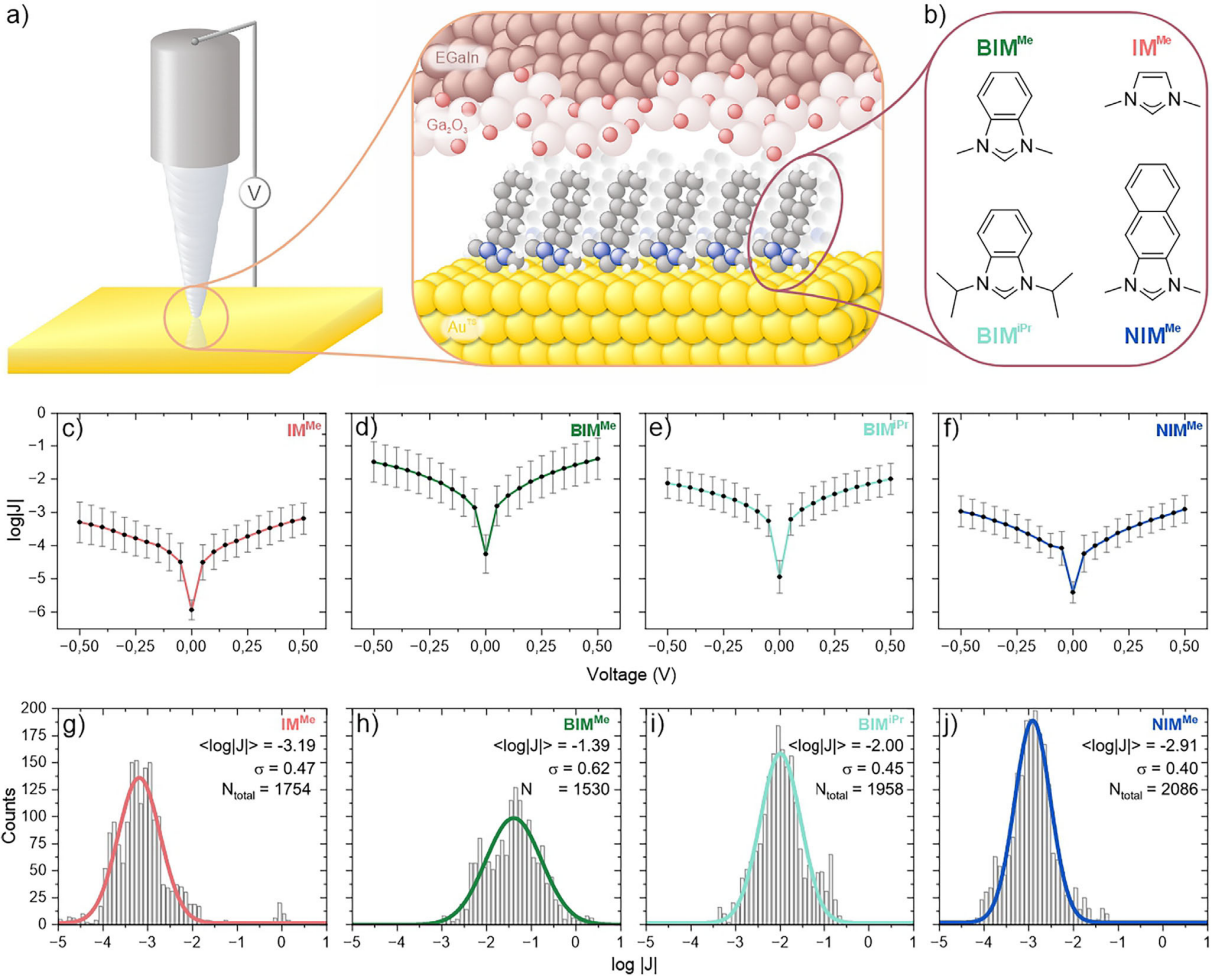
Summary of the EGaIn experiments. (a) Schematic presentation of the EGaIn junction and (b) the NHC molecules used for this study together with respective acronyms. (c–f) *log*|*J*(V)| plot derived from the average of all the *J*(V) traces (∼1500‐2000) obtained from 60–90 junctions. (g–j) Histograms of *log*|*J*(+0.5 V)| fitted with a Gaussian function to obtain the mean value and standard deviations indicated as error bars in (c)‐(f), respectively.

Suppression of molecular conduction has been reported for molecular systems with DQI; this approach, however, is limited to particular molecular designs where multiple‐conduction pathways interfere inevitably increasing the molecular lengths usually to >1 nm [53, 54, 55, 56, 57, 58, 59]. Therefore, alternative approaches are needed that suppress currents in molecules well below 1 nm to meet with the increasing demand for device miniaturization whilst ensuring stability. Surface modification based on carbene chemistry yields extremely stable structures, but their potential in nanoscale electronics is controversial. For example, single‐molecule measurements with break junctions derived from a series of SCH_3_−(Ph)*_n_*−BIM^iPr^ complexes (*n* = 0, 1, and 2; Ph = phenyl; see Figure 1b for BIM^iPr^ moiety) revealed that these molecules support high tunneling rates consistent with non‐resonant tunneling [50]. In contrast, the respective EGaIn junctions [60, 61, 62, 63] based analysis of (Ph)*_n_*−BIM^iPr^ (*n* = 0, 1, and 2) SAMs [51] despite also consistent with non‐resonant tunneling as the main mechanism of charge transport, indicated that these molecules are very good insulators [37]. Although, as recently shown, the preparation method of NHC SAMs can lead to an improved monolayer packing densities and conductivities (factor ∼40) [52], this still cannot account for the large discrepancy in tunneling rates relative to junctions of alkanetiolate SAMs (widely considered as highly insulating) by up to 5 orders of magnitude. To establish the conducting or insulating character of NHC SAMs, and to correlate conductance with their structure, we carried out EGaIn based conductivity experiments, supported by density functional theory (DFT) calculations, on a series of NHC SAMs with controlled length of the aromatic acene‐like backbone ranging from ∼3.3 to 7.9 Å in length. Our results indicate that NHC SAMs are ultra‐thin aromatic films featuring extremely insulating properties.,

## 2 Results and Discussion

### 2.1 Conductivity Analysis by EGaIn Junction

As shown in Figure 1a, a schematic presentation of the EGaIn (eutectic alloy of gallium and indium) junction indicates the liquid structure of the GaIn bulk and the thin (∼0.7 nm) [63], naturally formed Ga_2_O_3_ layer, which is in direct contact with the NHC SAM supported by the bottom (Au) electrode. All NHC molecules used for SAM formation in our experiments (IM^Me^, BIM^Me^, BIM^iPr^, and NIM^Me^, schematically shown in Figure 1b) are chemically bonded to the Au electrode via the carbene carbon atom from the imidazolium unit (IM) and characterized by a different number *n* of phenyl rings attached to the IM moiety in the acene fashion (*n* = 0 for IM^Me^; *n* = 1 for BIM^Me^ and BIM^iPr^; *n* = 2 for NIM^Me^). We studied two different lengths of the nitrogen side groups which is either methyl (IM^Me^, BIM^Me^ or NIM^Me^) or isopropyl (BIM^iPr^). The SAMs were derived from the respective imidazolium salts using previously reported procedures (for synthesis details please see Supporting Information for Ref [38]). The structure of analyzed here series of NHC monolayers—including packing density, film thickness, molecular tilt relative to the substrate, as well as bonding and thermal stability—has been comprehensively characterized in our previous study using a combination of X‐ray photoelectron spectroscopy (XPS), near‐edge X‐ray absorption fine structure (NEXAFS) spectroscopy, secondary ion mass spectrometry (SIMS), and thermally controlled SIMS (TP‐SIMS) techniques [38]. This comprehensive structural characterization forms foundation of the present work, we include control XPS and NMR data shown in the Supporting Information (see sections 2 and 3) to confirm that the monolayers are consistent with those reported in [38]. In brief, the tilt angle of molecules in these structures (measured from the surface normal), was quantified by NEXAFS spectroscopy and exhibited values (21°^–^28°) [38], which are typical for aromatic SAMs on metal substrates [60, 64, 65, 66, 67]. We would like to mention that in contrast to NEXAFS spectroscopy, analysis of tilt angle in NHC‐SAMs based on different vibrational spectroscopies provided only qualitative information so far [36, 68, 69]. Former XPS analysis was also used for estimation of the respective molecular footprints, which for short methylene side groups (IM^Me^, BIM^Me^ or NIM^Me^) exhibit values (0.23–0.29 nm^2^/molecule) [38] comparable with densely packed aromatic SAMs on metals derived from thiols [64, 65, 66, 67]. As expected, application of the more bulky isopropyl side groups in the case of BIM^iPr^/Au leads to roughly doubled molecular footprint (∼0.46 nm^2^/molecule) [38]. The chemisorption via the carbene carbon atom of all analyzed NHC molecules in our study was confirmed by measured desorption energies (using TP‐SIMS) which are exceptionally high (∼1.9 eV) for BIM^Me^/Au and NIM^Me^/Au, in contrast to IM^Me^/Au and BIM^iPr^/Au, for which this parameter is comparable to aromatic thiols on gold (∼1.4 eV) [37, 38]. The results of the EGaIn junction‐based experiments are summarized in Figure 1c–f exhibiting the current density *J*(*V*) [A/cm^2^] measurements as a function of the applied voltage in the range from −0.5 to +0.5 V, which is most commonly used for such measurements [37, 60, 61, 62, 70, 71, 72, 73]. To gain significant statistics for these measurements, about 60–90 individual junctions were formed using at least 4 different samples for each type of NHC SAM. Thus, taking about 20 traces for each formed EGaIn junction, the total number of all analyzed *J*(V) traces was within the range of ∼1500–2000. The *log*|*J*(V)| functions presented in Figure 1c–f are the log‐average of all these measurements for each type of NHC SAM with error bars indicating the log‐standard deviation (σ_*log*_) for each analyzed voltage. We note that the obtained *log*|*J*(V)| functions are approximately symmetric in all cases, indicating lack of significant rectification effect.

**FIGURE 2:**
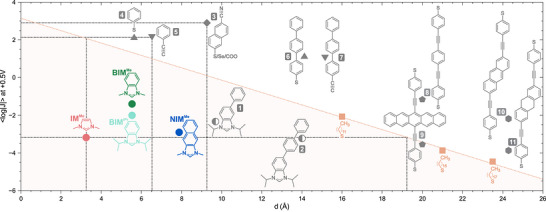
Plot of *log*|*J*(+0.5 *V*)| as a function of the length *d* of the molecule in the junction with the indicated measurement obtained for analysed here NHC SAMs (IM^Me^, BIM^Me^, BIM^iPr^, and NIM^Me^). The orange line indicates plot of the linear function (eq. 1) fitted to the measured reference SAMs based on alkanethiols (DDT, HDT, and ODT indicated by orange squares). For reference, the following data points measured by the EGaIn junction are shown: (1) BIM^iPr^‐Ph [51], (2) BIM^iPr^‐Ph_2_ [51], (3) NC‐NapS/Se/COO [60], (4) S‐Ph [62], (5) C≡CPh [62], (6) S‐Ph_3_ [62], (7) C≡C‐Ph_3_ [62], (8) OPE3 [54], (9) PC [54], (10) AC [55], and (11) AH [55], (see text for details).

To enable comparison of conductivity for analyzed here NHC monolayers with other SAMs, we used *log*|*J*(V)| values obtained at V = +0.5 V and Figure 1g–j shows these data in the form of histograms. The Gaussian functions fitted to these data were used to estimate both the mean value 〈*log*|*J*(+0.5 V)|〉 and its standard deviation (σ_*log*_). To compare our results with those obtained from other relevant systems, in Figure 2 we plotted obtained 〈*log*|*J*(+0.5 V)|〉 values as a function of the length (*d*) of the SAM‐forming molecules (measured from the anchoring atom to the most distal atom of the molecule). As a control, we also conducted measurements, and respective analysis (see Figure S6), for three selected alkanethiol‐based SAMs on Au (CH_3_‐(CH_2_)*_n‐1_*‐S/Au, *n* = 12 (DDT), 16 (HDT), and 18 (ODT)), which are also indicated in Figure 2. Using a general equation for coherent tunneling through a rectangular barrier, these data points in Figure 2 were fitted to Equation (1):

(1) $$\;\log\left(J\right)=\log\left(J_{0}\right)\;-\beta \frac{d}{2.303}$$

where *J*_0_ and β correspond to the injection current and the attenuation factor, respectively. The obtained value of β ≈ 0.77 Å^−1^ ($\approx 0.95\;n_{c}^{-1},$where *n*_c_ is the number of carbon atoms in aliphatic chain) obtained from these control experiments is in excellent agreement with former EGaIn based analysis of this parameter ($∼0.75\;{Å}^{-1}/(∼1.00\;n_{c}^{-1}$) [72, 74, 75] obtained using full series of alkanethiols on Au substrate. The corresponding log (*J*_0_) ≈ 2.8 is also within the typical values reported for alkanethiols in the EGaIn based experiments (∼2.0−4.0), where the exact value depends on the real contact area [75, 76, 77, 78]. Taking this fitted line as a well‐defined reference conductivity for standard alkanethiol SAMs on Au (which we take as a standard for insulating SAMs) [20, 21, 62, 70, 71], we can make several interesting observations. First, we note that all analyzed NHC SAMs exhibit conductivity which is by a few orders of magnitude lower than the respective alkanethiolate SAMs with the same value of *d*. In particular, for the shortest IM^Me^/Au monolayer of ∼3.3 Å, this effect amounts to 5 orders of magnitude lower conductivity. In general, the analyzed here NHC SAMs also show ∼3‐4 orders of magnitude conductivity suppression relative to alkanethiolates in single molecule junctions, and other types of insulating molecules, including siloxane [21] (via Si–O bond) or even bicyclic molecular form of silicon where DQI was observed [20]. This is rather surprising result considering that the backbone of the NHC molecules used in our study can be considered as having aromatic character (including the imidazolium unit, Figure 1b) [78]. Thus, we conclude that the carbene SAMs bonded to Au via the carbene carbon are more insulating than other types of SAMs anchored by C−Au bonds, including SAMs derived from alkynes or alkanes [61, 62, 79]. As reference data concerning these observations we have included in Figure 2 reported EGaIn measurements for a variety of aromatic SAMs including naphthalene based thiols/selenols/carboxylates (NC‐NapS/Se/COO) [60] and phenylacetylene (C≡Ph) [62] which have very similar values of *d* as NIM^Me^ and BIM^Me^/BIM^iPr^ molecules, respectively. The conductance values are lower by a factor of ∼10^6^ for NIM^Me^ and ∼10^4^ for BIM^Me/iPr^, compared to the reference aromatic SAMs with a similar thickness, confirming the extraordinary insulating properties of NHC SAMs. Interestingly, the thickest NHC SAMs in our survey, i.e., the NIM^Me^/Au monolayers, have the same conductance (within the precision of our measurements) as the same junctions but with IM^Me^/Au SAMs that are only half as thick (*d*_NIM_ */d*_IM_ ∼ 2.5). This comparison shows that modification of the molecular backbone by adding a naphthalene unit to imidazolium moiety in acene fashion must lead to significantly improved conductivity of the whole system to compensate for doubling of its length. The improved conductivity upon adding an aromatic ring to the imidazolium moiety in acene fashion is also very well visible for BIM^Me^/Au monolayers which, despite an increase of the *d* by a factor ∼1.7 compared to IM^Me^/Au, exhibits a ∼60 times higher conductivity. As a reference concerning this effect we have also inserted in Figure 2 data obtained for junctions with BIM^iPr^−(Ph)*_n_* SAMs (*n* = 0, 1, and 2) in a oligophenyl fashion [51]. For this series, Yoon et al. found that the current decays exponentially with *d* with a value of β ≈ 0.48 Å^−1^ [51]. We note that conventional β‐parameter analysis is not applicable to the acene‐like series of NHC SAMs investigated herein. In contrast to oligophenyl systems, acenes exhibit a progressive narrowing of the HOMO–LUMO gap with increasing aromatic backbone length, resulting in a concomitant, length‐dependent modulation of the tunneling barrier height [80, 81, 82, 83].

The last but not least observation which we can make is comparing conductivity of BIM^Me^/Au and BIM^iPr^/Au monolayers, which in the former case is higher by a factor of ∼4. Assuming that conduction through every molecule is the same, the value of *J* should be proportional to the packing density of molecules in the junction, as has been experimentally demonstrated for alkanethiolate SAMs by Lindsay et al., [83]. Therefore, considering twice higher packing density for BIM^Me^/Au than BIM^iPr^/Au, the increase in relative conductivity by the factor of 2 is fully expected. The factor 2 discrepancy, however, can be explained by the different side groups on the nitrogen and their interaction with the metal substrate as indicated by our DFT calculations (see next section). For the sake of completion, we would like to point out that all monolayers have very similar surface energies (∼50 mJ/m^2^) measured by water contact angle [38]. We emphasize that template‐stripped gold substrates were used to support the SAMs, as this method yields ultrasmooth surfaces [84]. In addition, our previous combined STM and contact angle study demonstrated that NHC SAM surfaces are atomically smooth [37]. Given that all SAMs exhibit similar topography, thickness, tilt angles, and packing densities, we expect the carbene–top electrode interface to be comparable across the series. Consequently, the effective contact area with the EGaIn top electrode should be statistically equivalent for all SAMs, ruling out structural differences in the SAMs as the origin of the observed variations in current [85].

### 2.2 Transport Calculations

To gain detailed insight into the experimental observation, we performed transport calculations using the combined non‐equilibrium Green's function (NEGF) + density functional theory (DFT) approach as implemented in SIESTA and its TranSIESTA module [86, 87, 88] and computed the electronic transmission, *T*(*E*), for all four NHCs. Modelling an EGaIn electrode is challenging as it lacks a well‐defined crystalline structure [88]. However, previous studies suggest that replacing the EGaIn electrode with gold can provide meaningful insights into SAM‐based junctions [53, 89]. Here, it is important to note that the actual SAM‐EGaIn interface is expected to have different tunnelling barriers and coupling strength than SAM‐Au surface. SAM systems can be effectively modelled using a single molecule in a unit cell when intermolecular interactions are minimal and have a negligible impact on the transmission [53, 89]. However, in certain cases, the effect of intermolecular interaction on transmission is substantial [58].

**FIGURE 3:**
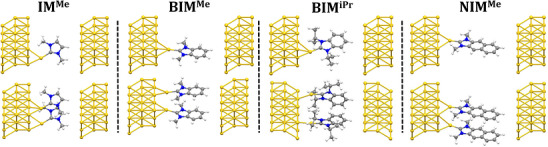
Side‐view of the optimized structures of all four NHCs in Au‐*n*NHC‐Au (*n* = 1, 2) junction. The top and bottom panels display *n* = 1 NHC and *n* = 2 NHCs per 3 × 3 Au(111) unit cell, respectively. H, C, N, and Au are represented by white‐, grey‐, blue‐, and yellow‐colored balls, respectively.

To begin with and to investigate the effect of intermolecular interaction on electron transport, we analyzed Au−*n*NHC‐Au (*n* = 1, 2) junctions where single (*n* = 1) and two (*n* = 2) NHCs positioned between 3 × 3 Au (111) left and right electrodes, with an Au adatom interacting with the carbene carbon (see Figures 3 and S7 for side and top view of the optimized junction respectively). The adatom was considered in light of earlier reports on similar NHCs and to maintain a consistent binding mode across all NHC junctions [90, 91, 92]. The stacking distances (*i.e*., shortest atom‐to‐atom distance excluding H) in the Au−1NHC−Au and Au−2NHC−Au junctions are 7.3 Å, 3.5 Å for IM^Me^; 8.1 Å, 3.3 Å for BIM^Me^; 8.6 Å, 3.2 Å BIM^iPr^; and 7.9 Å, 3.4 Å for NIM^Me^, respectively. In Au−2NHC−Au, these distances fall within the typical π‐stacking range. Here it is worth noting that, earlier studies demonstrate that NHCs bind to Au surfaces via strong σ‐donation from the carbene carbon lone pair into the metal d‐orbitals, with negligible π‐back donation. This results in a strong metal─carbon bond, in contrast to the Au−S interaction in thiols, which involves different interfacial hybridization and charge redistribution. Consequently, depending on the side‐groups and molecular backbone, NHC anchoring leads to stronger binding and enhanced chemical stability compared to conventional thiol linkers [27, 28]. This distinct interfacial electronic structure is expected to influence charge transport by modifying molecule−electrode coupling and energy level alignment.

**FIGURE 4:**
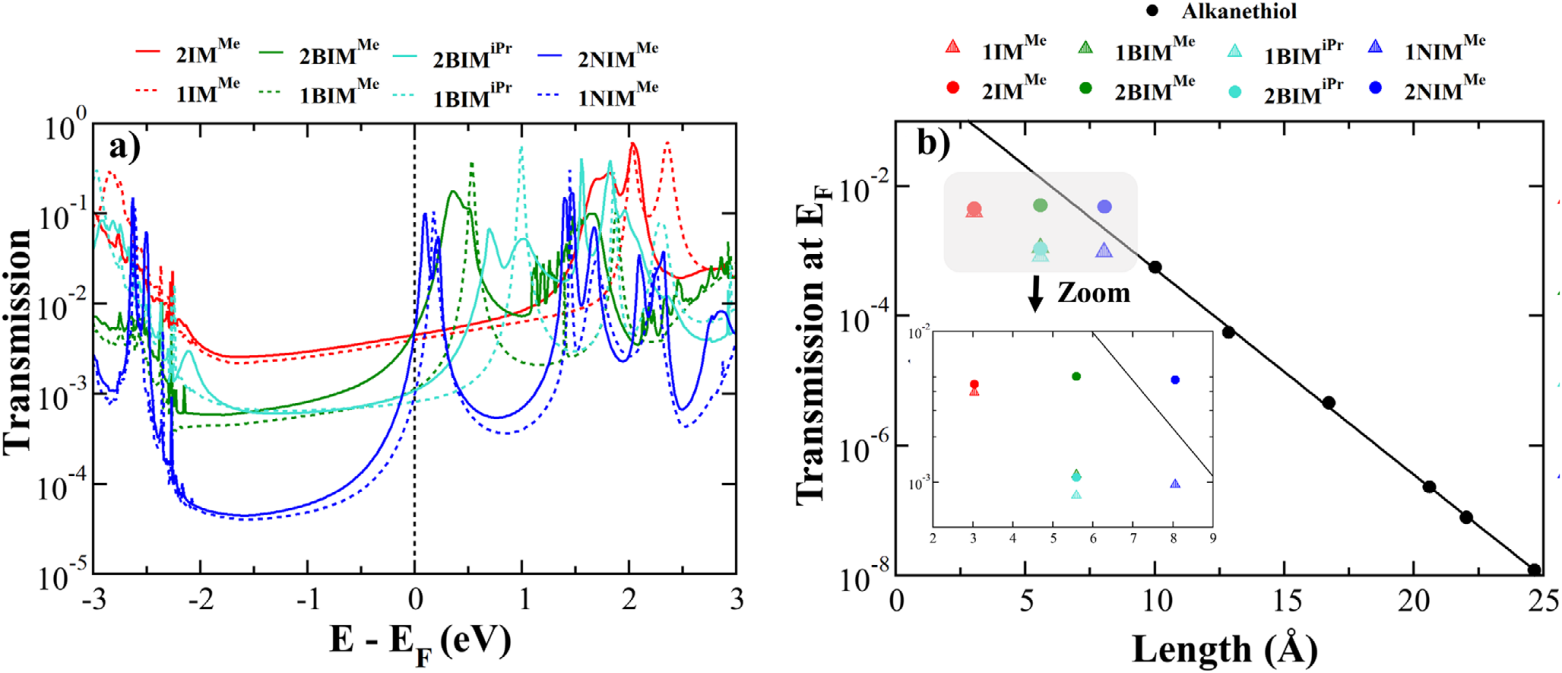
Calculated transmission function per molecule of Au−*n*NHC−Au (*n* = 1, 2) junction as a function of energy relative to the *E*_F_ (a) and transmission at *E*_F_ plotted against molecular length, for all four NHCs with alkanethiols as a reference (b).

The transmission per molecule for all Au−*n*NHC−Au systems is plotted in Figure 4a. The proximity of the lowest unoccupied molecular orbital (LUMO) peak to the Fermi energy (*E*_F_) in the transmission plot indicates LUMO‐dominated electron transport at low bias. It is evident from the plot that π‐stacking interaction between molecules shifts the energy of the LUMO (*E*_L_) towards *E*_F_, enhancing transmission at *E_F_*, *T*(*E*_F_), and hence the conductance for Au−2NHC−Au as compared to Au−1NHC−Au across all NHCs. Previous studies have similarly shown that the π‐stacking interaction increases molecular conductance in a similar π‐conjugated, *N*‐containing heterocyclic system [93]. The LUMO peak shifts closer to *E_F_* (*i.e., E_L_* [IM^Me^] > *E*_L_[BIM^Me^] > *E*_L_[NIM^Me^]) with increasing length of the NHCs (i.e., IM^Me^ < BIM^Me^ < NIM^Me^), consistent with the stabilization of *E*_L_ as conjugation extends (see Table S1). In general, transmission decreases with increasing molecular length, as observed in alkanethiols and oligophenyls [62, 72, 74, 75]. However, shift of *E*_L_ towards *E*_F_ due to extended conjugation enhances transmission as it is observed for acenes. The interplay between these opposing effects determines the conductance (*i.e., T*(*E*_F_) at zero bias) trend across the NHC series considered in this study. Consequently, despite its longer molecular length, NIM^Me^ exhibit *T*(*E*_F_) comparable to IM^Me^ consistent with experimental observation.

For NHCs of the same length (BIM^Me^ and BIM^iPr^), the frontier molecular orbital (FMO) energies of the isolated molecule are similar, with only a 0.05 eV difference in Δ*E*_H − L_ (see Table S1). In the junction, however, the interaction between the bulkier side groups at the N atom and Au−surface destabilize the LUMO in BIM^iPr^, shifting it further away from *E*_F_. This results in a lower *T*(*E*_F_) for BIM^iPr^ compared to BIM^Me^ which is also consistent with our experimental observation. *T*(*E*_F_) for all Au−*n*NHC−Au junctions are plotted against molecular length and compared with alkanethiols (see Figure 4b). *T*(*E*_F_) of all the NHCs lie below the alkanethiol reference line, except for the Au−2NIM^Me^−Au, where LUMO peak near the *E_F_* significantly enhances the transmission. The calculated *T*(*E*_F_) values for Au−NHC−Au fall within the 10^−3^ − 10^−4^ range (see Table S2). We also checked the influence of work function, finite bias, ionization and bonding configuration (without the adatom), and see that LUMO peak ordering remains the same for all conditions but its position relative to *E_F_* significantly influences the *T*(*E_F_*) and hence conductance. Respective data (Figures S8–S15) and detailed discussions concerning these influences are included in the Supporting Information. Interestingly, the application of finite positive bias shifts LUMO peak further away from the bias window, leading to a decrease in *T*(*E*) around *E*_F_ for all the NHCs, which may be responsible for the observed insulating behavior (see Figure S16). This is attributed to the combined effect of interfacial electronic coupling, dipole formation at the interface, orbital alignment with respect to the electrode Fermi level, and bias‐induced polarization in the junction.

## 3 Conclusions

To conclude, our EGaIn based experiments conducted for the series of well‐defined aromatic NHC SAMs on Au(111) demonstrate, in all cases, that the conductivity is by 3–5 orders of magnitude lower compared to the standard insulating aliphatic SAMs formed by the molecules of the same length. By using NHC molecules featuring different length of the molecular acene‐like backbone, we show that contrary to aliphatic [72, 74, 75] or oligophenyl [51, 62] based SAMs, the increased length of such backbone, does not change or even increase conductivity of the respective monolayer. Our data also show that increased size of the nitrogen side groups lowers conductivity more than expected considering reduced packing density, indicating thus the impact of the intermolecular interactions on molecular conductance. The DFT+NEGF calculations, incorporating intermolecular interactions, bonding configuration (with and without the adatom), electrode work function, finite positive bias, and ionization, provide a qualitative framework to understand the experimental trends. Moreover, our theoretical analysis gives no indication that suppressed conductance of the NHC SAMs is related to the DQI effects [57, 58, 59], reported also in EGaIn experiments [53, 54, 55, 56]. Our calculations indicate that exceptionally high conductance suppression in NHC SAMs with imidazolium moiety can be explained by the effective shift of the LUMO relative to the bias window at a finite positive bias voltage. Considering that strong suppression has also been found for oligophenyl‐like NHC SAMs (Figure 2) [51], it seems that monolayers derived from imidazolium as a bonding group are good insulators. We stress at this point that former EGaIn based experimental study comparing conductivity of analogous SAMs (aromatic and aliphatic) with different anchoring groups such as thiols, selenols, carboxylic acids or even alkynes demonstrated no significant change in their conductivity [60, 61, 62, 73], making imidazolium based NHC SAMs particularly interesting system. Bonding via the carbene atom—embedded within the cyclic imidazolium unit—is structurally distinct from typical anchoring groups used in SAMs, which usually involve single atoms or small functional groups mentioned above. To the best of our knowledge, imidazolium‐based systems represent the first class of SAMs in which the surface anchoring atom is incorporated directly into an aromatic ring, resulting in a fundamentally different bonding geometry. Whether this bonding motif itself may influence charge transport remains an open question and will require further experimental investigation, which we plan to pursue in future studies. We note that suppression of SAMs conductivity via selection of the proper bonding group is conceptually simpler and gives much more flexibility in design as compared to DQI‐based conductivity suppression, where DQI effects have to be controlled by carefully selected design and usually demand using longer molecules [50]. Thus, current work opens up the possibility of using NHC SAMs with imidazolium bonding moiety as an ultra‐thin, and exceptionally insulating, aromatic films which could be used for molecular/organic electronics applications including functionalization of the gate electrodes in organic field emission transistors (OFET). In particular, the longest member of the analyzed here NHC series (NIM^Me^), which exhibits also high thermal stability and packing density [38], would be of particular interest.

## Author Contributions

The manuscript was written through contributions of all authors. All authors have given approval to the final version of the manuscript.

## Funding

This study was supported by the National Science Centre Poland (grant DEC‐2018/31/B/ST5/00057), The Polish National Agency for Academic Exchange (stipend BPN/BEK/2022/1/00339), ERC Grant—QLIMIT (Grant Agreement No 865870). This work is supported by the Dutch Research Council (NWO), VI.C.222.037.

## Conflicts of Interest

The authors declare no conflict of interest.

## Supporting information




**Supporting File 1**: Additional EGaIn data, DFT analysis description and additional data, Figures S1–S13 and Tables S1, S2, indicated in the manuscript.

**Supporting File 2**: anie73017‐sup‐0002‐SuppMat.zip.

## Data Availability

The data that support the findings of this study are available from the corresponding author upon reasonable request.
