# Supplementary material for: Ultra‐Thin and Highly Insulating Aromatic Monolayers by N‐Heterocyclic Carbenes
Source: Angew Chem Int Ed Engl. 2026 Jun 10;65(33):e4614418. doi: 10.1002/anie.4614418 (PMC13452479; doi:10.1002/anie.4614418)
Supplement: Supplementary file 1 [file ANIE-65-e4614418-s002.pdf]

Supporting Information

for

Ultra-Thin and Highly Insulating Aromatic Monolayers by N-Heterocyclic Carbenes

Mateusz Wróbel,[1] Raka Ahmed,[2] William Bro-Jørgensen,[2] Krzysztof Kozieł,[3] Christian A. Nijhuis,[4] Gemma C. Solomon,[2,5]* and Piotr Cyganik[1]**

[1] Faculty of Physics, Astronomy and Applied Computer Science, Smoluchowski Institute of Physics, Jagiellonian University, 30-348 Krakow, Poland.

[2] Department of Chemistry and Nano-Science Center, University of Copenhagen, Copenhagen 2100, Denmark.

[3] Faculty of Chemistry, Jagiellonian University, 30-387 Krakow, Poland.

[4] Department of Molecules and Materials, MESA+ Institute for Nanotechnology, Molecules Center and Center for Brain-Inspired Nano Systems, Faculty of Science and Technology, University of Twente, Enschede 7500 AE, The Netherlands.

[5] NNF Quantum Computing Programme, Niels Bohr Institute, University of Copenhagen, DK-2100 Copenhagen, Denmark

Supplementary Materials

1. Synthetic protocols 3

1.1. General considerations 3

2. NMR analysis 3

2.1. Measurement details 3

2.2. NMR spectra 5

2.2.1. 1,3-dimethyl-1*H*-imidazol-3-ium hydrogen carbonate (IM^{Me}) 5

2.2.2. 1,3-dimethyl-1*H*-benzo[*d*]imidazol-3-ium hydrogen carbonate (BIM^{Me}) 6

2.2.3. 1,3-diisopropyl-1*H*-benzo[*d*]imidazol-3-ium hydrogen carbonate (BIM^{iPr}) 7

2.2.4. 1,3-dimethyl-1*H*-naphtho[2,3-*d*]imidazol-3-ium hydrogen carbonate (NIM^{Me}) 8

3. Sample preparation 9

4. XPS analysis 9

4.1. Measurement details 9

4.2. XPS spectra 10

4.3. Analysis 10

4.4. Calculation of the SAM thickness 11

5. EGaIn analysis 12

5.1. Measurement details 12

5.2. Data for alkanethiols 12

6. Computational details 13

6.1. Transport calculations 13

6.2. Calculation at wide band limit (WBL) 14

6.3. Discussion of charge transport calculations 15

7. References 24

1. Synthetic protocols

1.1. General considerations

In this study, we used NHC precursors in the form of a hydrogen carbonate salts. Detailed synthetic protocols are provided in the Supplementary Material of our previous work.[1] In short, all of the SAM-forming NHC compounds, namely 1,3-dimethyl-1*H*-imidazol-3-ium hydrogen carbonate (IM^{Me}), 1,3-dimethyl-1*H*-benzo[*d*]imidazol-3-ium hydrogen carbonate (BIM^{Me}), 1,3-diisopropyl-1*H*-benzo[*d*]imidazol-3-ium hydrogen carbonate (BIM^{iPr}), and 1,3-dimethyl-1*H*-naphtho[2,3-*d*]imidazol-3-ium hydrogen carbonate (NIM^{Me}), were obtained through *N*-alkylation of corresponding imidazoles with alkyl iodides, and converted to hydrogen carbonate salts by use of ion-exchange resin. The corresponding ^{1}H and ^{13}C NMR spectra (see **Figures S1-S4**) show that the compounds are pure and identical to those previously reported.[1] Successful removal of the iodine from the compounds was confirmed by measuring XPS of the prepared samples (see **Figure S5c**). Alkanethiols, namely 1-dodecanethiol (DDT, Alfa Aesar), 1-hexadecanethiol (HDT, Alfa Aesar), and 1-octadecanethiol (ODT, Sigma Aldrich) were used as received without further purification. Absolute ethanol (VWR Chemicals) was used as a solvent, with prior degassing using nitrogen flow for about 1h.

2. NMR analysis

2.1. Measurement details

The NMR spectra for the NHC compounds (see **Figures S1-S4**) were acquired on a Bruker Avance III 600 MHz spectrometer. Chemical shifts are reported in delta (δ) units, expressed in parts per million (ppm) downfield from the internal standard – tetramethylsilane or relatively to the solvent residual peak. The proton signals for H-C(2) and the bicarbonate anion were not detected due to rapid exchange with the deuterium from the solvent (D_2O). In the NMR spectra,

the C(2) signal was obscured by signal splitting arising from $^{1}J_{C\text{-}D}$ coupling. Additionally, the signal for the bicarbonate carbon was not observed, which may be attributed to its long relaxation time (T1) and the lack of Nuclear Overhauser Effect (NOE) enhancement. The NMR data for all NHC compounds are in agreement with the data previously reported.[1]

2.2. NMR spectra

2.2.1. 1,3-dimethyl-1*H*-imidazol-3-ium hydrogen carbonate (IM^{Me})

^{1}H NMR (600 MHz, D_2O) δ = 7.42 (s, 2H), 3.89 (6, 3H). ^{13}C NMR (151 MHz, D_2O) δ 126.35, 38.57.

a)

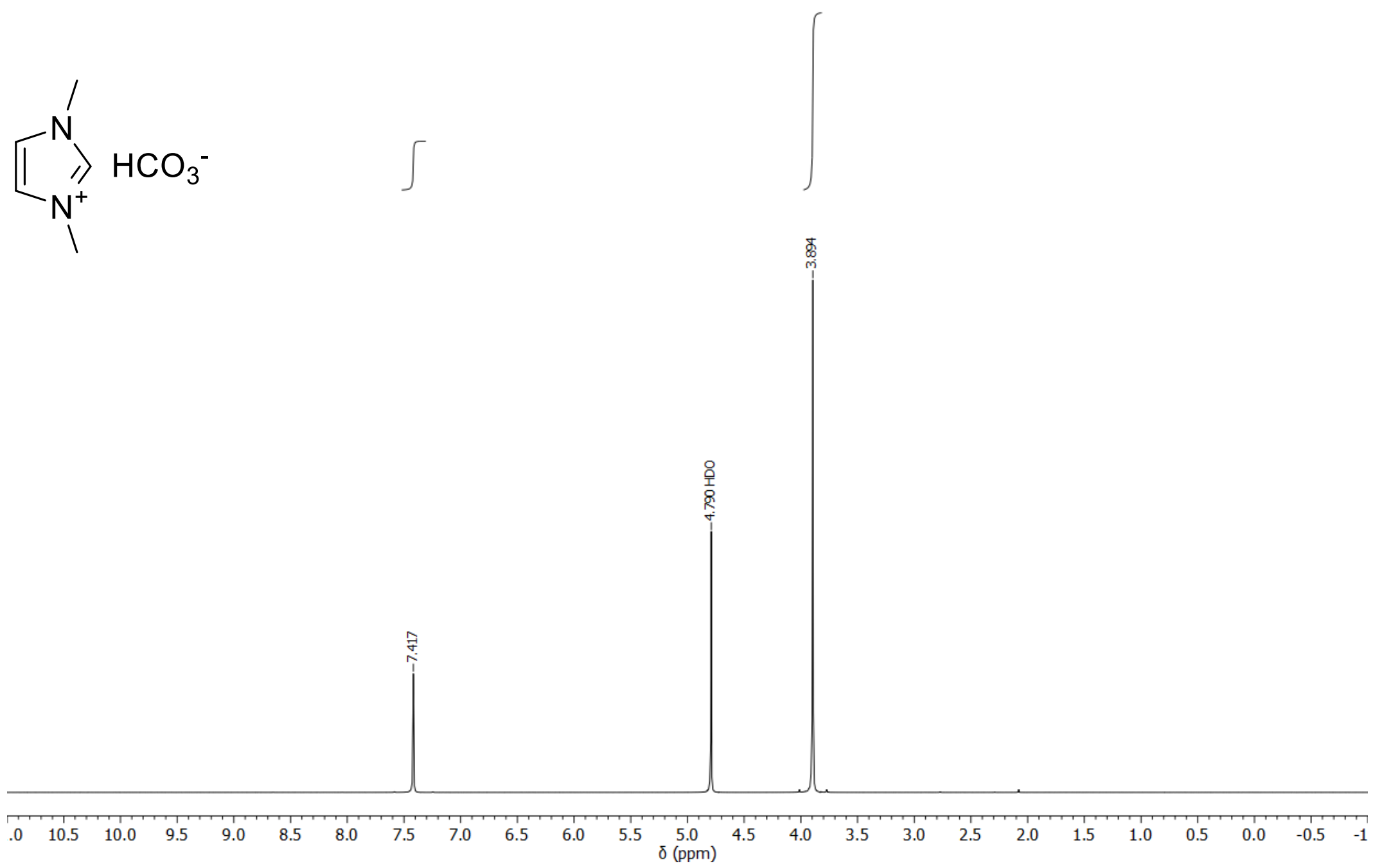

b)

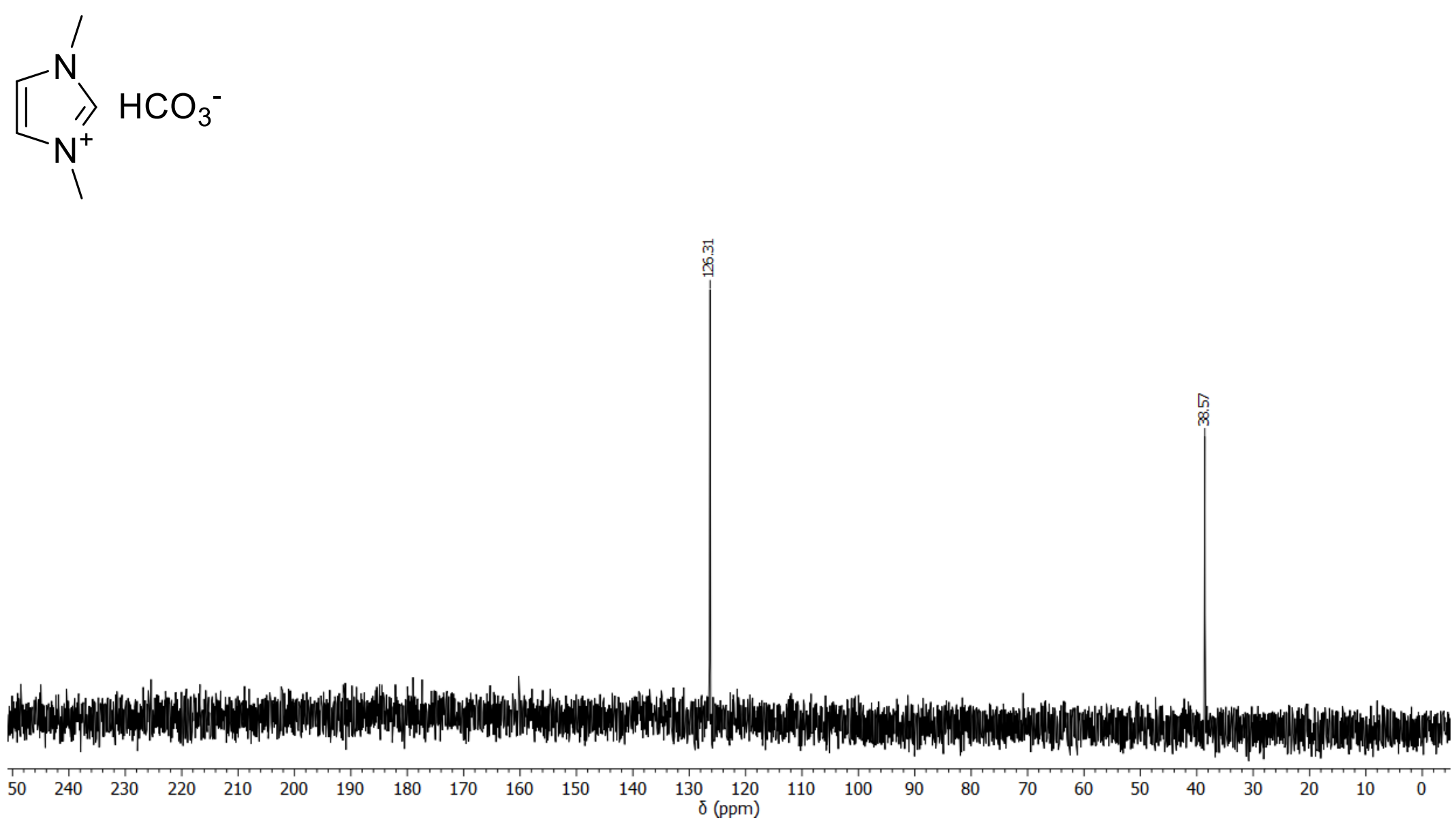

Figure S1. ^{1}H (a) and ^{13}C (b) NMR spectra for 1,3-dimethyl-1*H*-imidazol-3-ium hydrogen carbonate (IM^{Me}).

2.2.2. 1,3-dimethyl-1*H*-benzo[*d*]imidazol-3-ium hydrogen carbonate (BIM^{Me})

^{1}H NMR (600 MHz, D_2O) δ = 7.85 (dd, *J*=6.3, 3.1, 2H), 7.71 (dd, *J*=6.3, 3.1, 2H), 4.10 (s, 6H).

^{13}C NMR (151 MHz, D_2O) δ = 133.05, 127.91, 113.97, 33.91.

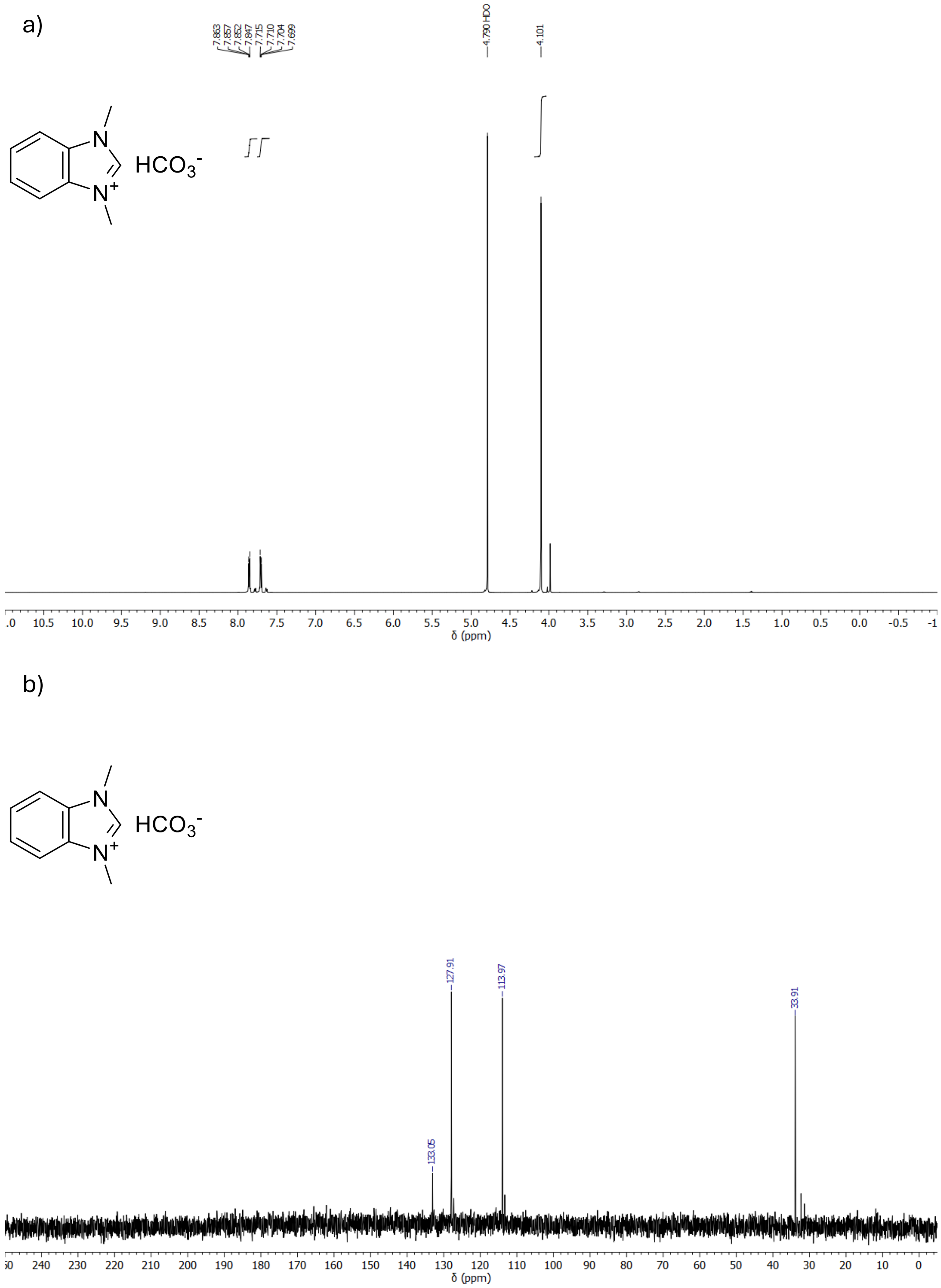

Figure S2. ^{1}H (a) and ^{13}C (b) NMR spectra for 1,3-dimethyl-1*H*-benzo[*d*]imidazol-3-ium hydrogen carbonate (BIM^{Me}).

2.2.3. 1,3-diisopropyl-1*H*-benzo[*d*]imidazol-3-ium hydrogen carbonate (BIM^{iPr})

^{1}H NMR (600 MHz, D_2O) δ = 7.93 (dd, *J*=6.3, 3.1, 2H), 7.67 (dd, *J*=6.5, 3.0, 2H), 5.04 – 4.96 (m, 2H), 1.68 (d, *J*=6.8, 12H). ^{13}C NMR (151 MHz, D_2O) δ = 131.55, 127.03, 114.01, 51.55, 21.51.

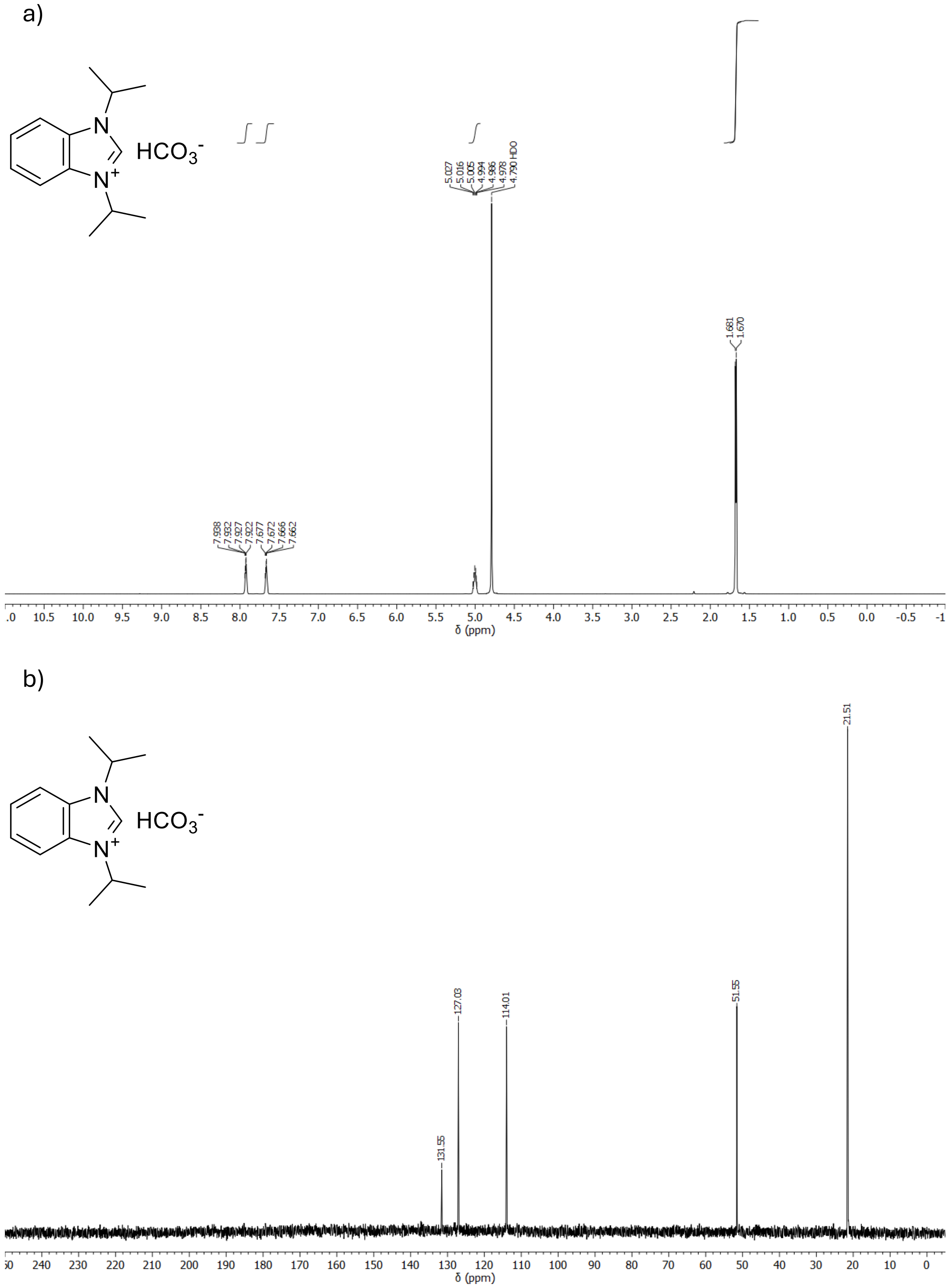

Figure S3. ^{1}H (a) and ^{13}C (b) NMR spectra for 1,3-diisopropyl-1*H*-benzo[*d*]imidazol-3-ium hydrogen carbonate (BIM^{iPr})

2.2.4. 1,3-dimethyl-1*H*-naphtho[2,3-*d*]imidazol-3-ium hydrogen carbonate (NIM^{Me})

^{1}H NMR (600 MHz, DMSO-d_6) δ = 9.86 (s, 1H), 8.61 (s, 2H), 8.22 (dd, *J*=6.3, 3.3, 2H), 7.68 (dd, *J*=6.5, 3.2, 2H), 4.16 (s, 6H). ^{13}C NMR (151 MHz, DMSO-d_6) δ = 147.19, 130.87, 130.81, 128.20, 126.51, 110.69, 33.25.

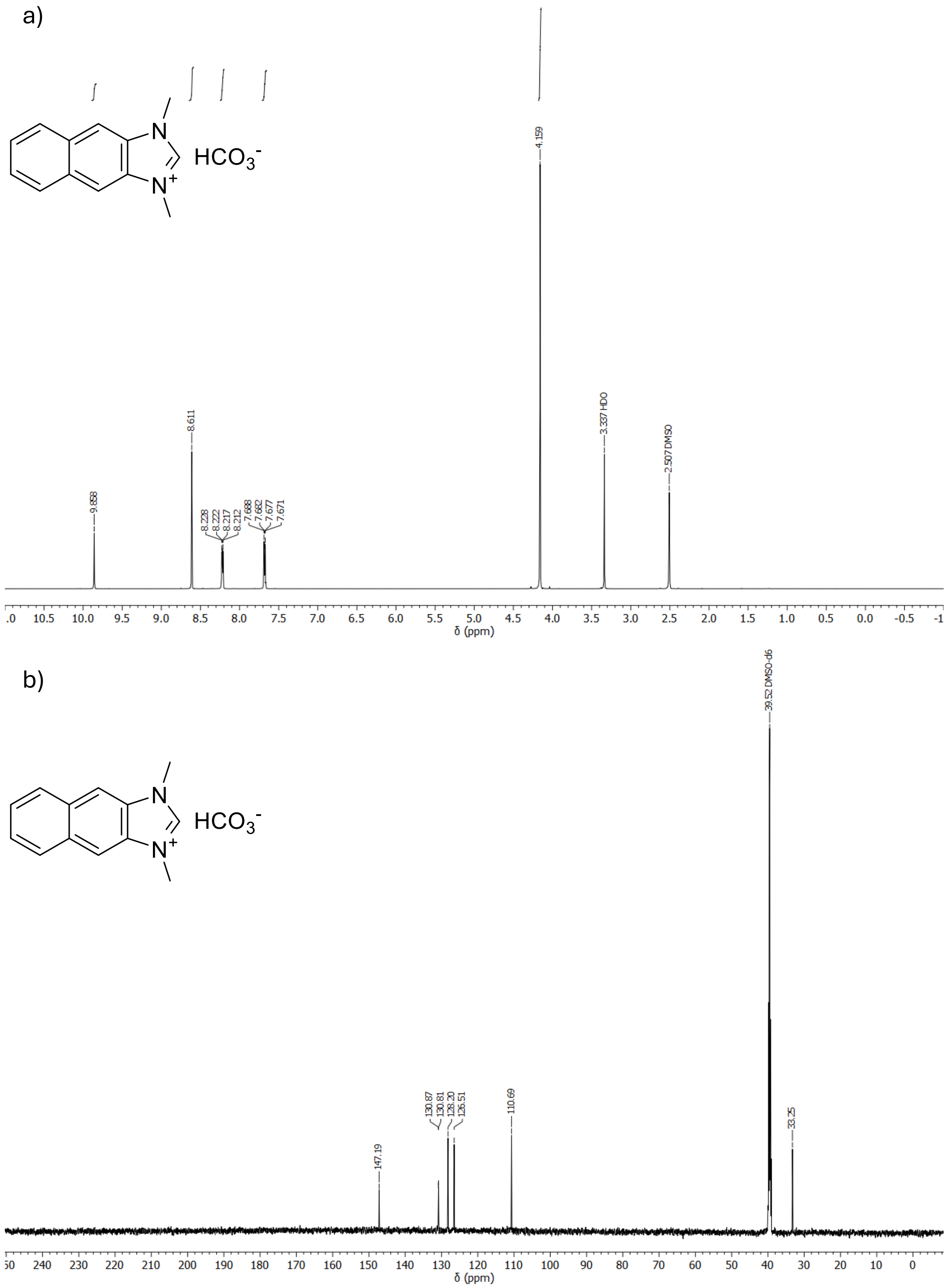

Figure S4. ^{1}H (a) and ^{13}C (b) NMR spectra 1,3-dimethyl-1*H*-naphtho[2,3-*d*]imidazol-3-ium hydrogen carbonate (NIM^{Me}**)**

3. Sample preparation

Ultra-flat gold substrates were formed using a well-established method.[2] First, a gold layer of approx. 200 nm thickness was deposited on a monocrystalline silicon (Si-100) wafer using an e-beam evaporator. Glass slides (1.0 x 1.5 cm^2) were washed and sonicated with deionized water, acetone, isopropanol, and dried using a nitrogen stream. An optical adhesive (Norland Optical Adhesive NOA 61) was used to glue glass stripes to the gold layer on a silicon wafer by UV curing (bluepoint 2 easycure, Dr. Hönle AG UV Technology) for 30 min. Afterwards, the substrates were aged for at least 1 day in ambient conditions to obtain optimum adhesion to glass slides. All SAMs (both NHCs and alkanethiols) were formed by the immediate transfer of glass slides, freshly cleaved using a razor blade, to the corresponding 1 mM ethanolic solutions of SAM-forming compounds (, and an overnight (~18 h) incubation at room temperature. The whole procedure (cleaving, incubation) was performed in a nitrogen-filled glovebox (MBraun, <5 ppm O_2, and <5 ppm H_2O). After incubation, the samples were removed from the solutions, washed with copious amounts of absolute ethanol, and gently dried under a nitrogen stream. All EGaIn measurements were performed immediately after sample preparation.

4. XPS analysis

4.1. Measurement details

Before conducting electrical characterization of the NHC SAMs, all samples were measured using X-ray photoelectron spectroscopy (XPS) to verify the formation of proper monolayers (see **Figure S5**). To obtain the spectra, the ThermoFisher Scientific ESCALAB™ QXi Microprobe workstation with a monochromatized Al Kα X-ray source (E = 1486.6 eV) and a 650 μm spot size was used. Measurements were performed with a base pressure below 5×10^{-10} mbar. The spectra were acquired in the normal emission geometry with 0.1 eV energy step,

using the Au $4f_{7/2}$ peak (84.0eV) for the calibration of the binding energy (BE) scale. For data collection and processing, the Thermo Scientific Avantage® software was used. Individual peaks were fitted with pseudo-Voigt profiles.

4.2. XPS spectra

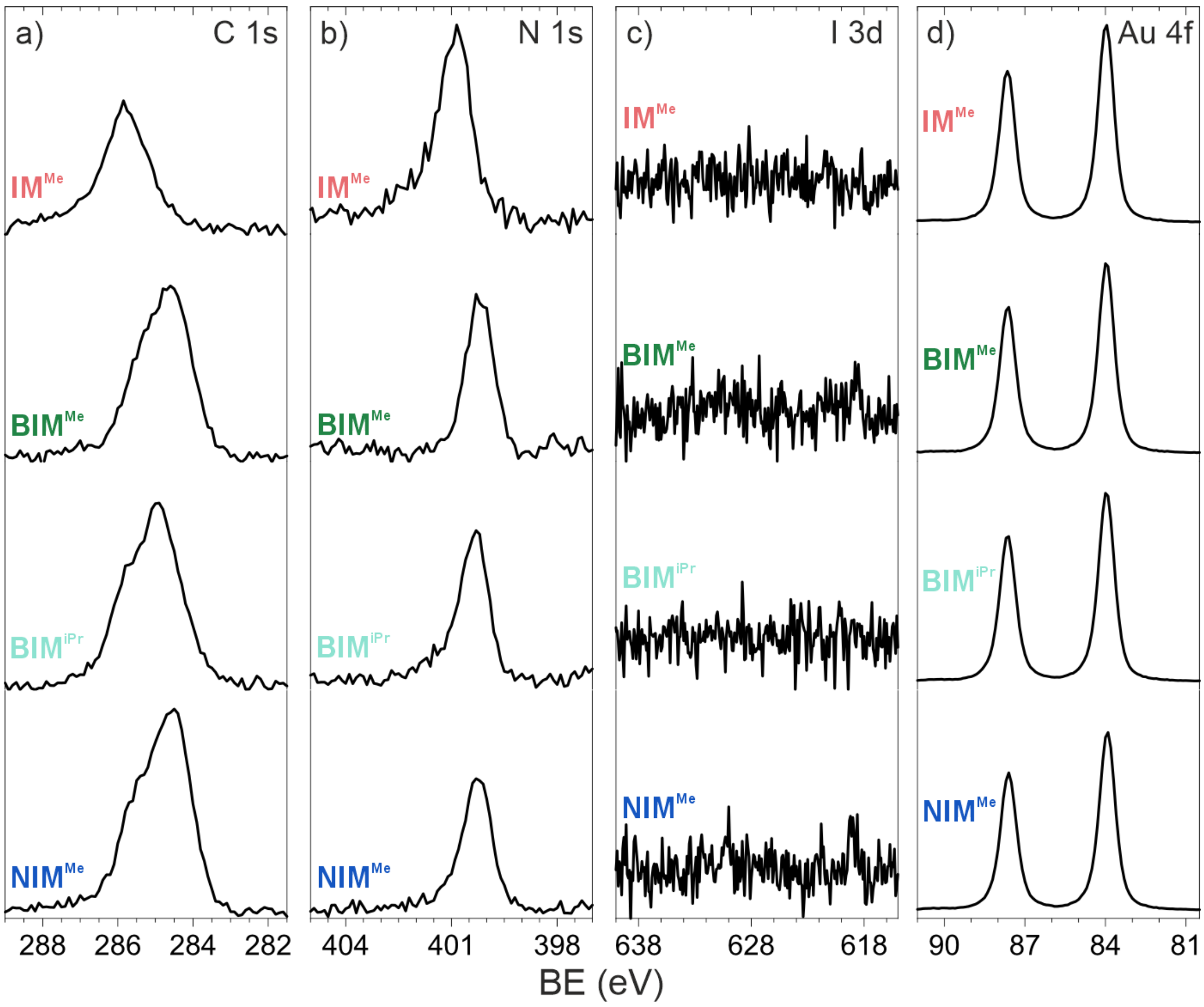

Figure S5. XPS analysis of the NHC SAMs. (a-d) C 1s (a), N 1s (b), I 3d (c), and Au 4f (d) spectra of the NHC SAMs.

4.3. Analysis

The C 1s spectrum for the IM^{Me} exhibits a single symmetric peak at the binding energy (BE) of ~286.0 eV. For other molecules, the C 1s peak becomes asymmetric, and two components can be fitted at BE of ~285.5 eV and ~284.5 eV, corresponding to the imidazolium unit and benzene

rings and bulky side groups in BIM^{iPr}, respectively. The N 1s spectra exhibit a single component at ~400.9 eV for IM^{Me} and at ~400.3 eV for the rest of the molecules in the series. No traces of iodine contamination were observed, as confirmed by dedicated measurements in the I 3d region. This is important information considering difficulties reported recently with obtaining iodine-free NHC monolayers using hydrogen carbonate salts.[3] The positions of peaks and relative intensities are in agreement with data previously reported for the NHC molecules deposited on the gold surface.[1]

4.4. Calculation of the SAM thickness

Based on the C 1s and Au 4f signal intensities (I_{C1s} and I_{Au4f}, respectively), the effective thicknesses (d) of the monolayers were calculated using the equation:

$$\frac{I_{C1s}}{I_{Au4f}} = K\,\frac{1 - \exp\left(-\frac{d-z}{\lambda_{C1s}}\right)}{exp\left(-\frac{d}{\lambda_{Au4f}}\right)},$$

where λ_i is the attenuation length for C 1s / Au 4f signal (28 Å and 31 Å, respectively), z is the distance from the gold surface to the first carbon atom (2.1 Å for NHCs[4]) and K is a constant depending on the equipment specification. The attenuation lengths λ were calculated using the standard formula: $\lambda = 0.3\,{E_K}^{0.64}$, where the E_K is the kinetic energy of electrons[5]. The K constant was determined to be 0.079 using the 1-dodecanethiol on the gold surface as a reference. The obtained effective thicknesses for NHC molecules are 8.1(0.4) Å, 8.9(0.4) Å, 9.2(0.5) Å, and 10.7(0.5) Å for IM^{Me}, BIM^{Me}, BIM^{iPr}, and NIM^{Me}. The errors were estimated as ~5% of the values. These data are in agreement with data reported by us before

,[1] and, therefore, we conclude that our monolayers are of the same quality.

5. EGaIn analysis

5.1. Measurement details

Cone-shaped tips of eutectic gallium-indium alloy (EGaIn) were used as a top electrode to collect current-voltage curves using procedures described previously.[6,7] In short, 60-90 junctions were made on 4-5 samples of each SAM (around 15 junctions per sample), with each tip used up to 4 times and not exceeding the geometric contact area of around 500 μm^2. A bias of ± 0.5 V was applied to the tips (sample grounded) in the following manner: 0 V → +0.5 V → 0 V → -0.5 V → 0 V, with a step of 0.05 V. Prior to collecting 20 current density-voltage scans, the tips were stabilized for couple of second at +0.5 V bias in contact with samples. The collected data were analyzed using publicly available code Gaussfit.[8] Gaussian mean values and respective standard deviations (σ) were used to construct J(V) plots.

5.2. Data for alkanethiols

A series of alkanethiols with n = 12, 16, and 18 (dodecanethiol, hexadecanethiol, and octadecanethiol, respectively) was chosen as reference systems with well-established conductivity.[9–11] The measurement protocol was the same as for the NHC samples, with 30-60 junctions made on 2-3 samples for each alkanethiol. The summary of measurements is presented in **Figure S6**.

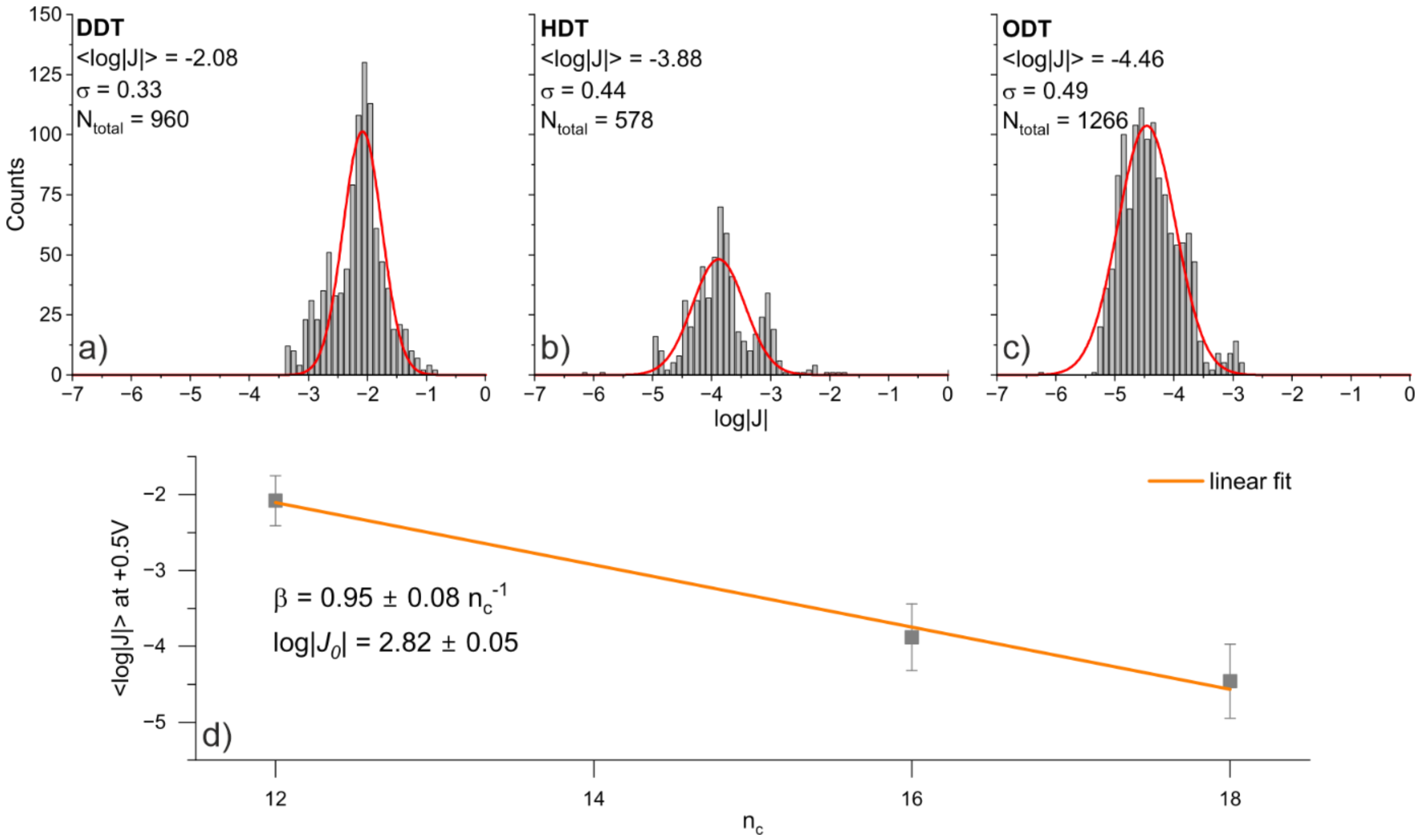

Figure S6. Summary of EGaIn measurements for alkanethiols. (a)-(c) Histograms of $log|J(+0.5V)|$ fitted with a Gaussian function to obtain the mean value and standard deviations indicated as error bars in (d). (d) Plot of $\langle log|J(+0.5V)|\rangle$ as a function of the number n_C of carbon atoms in the alkanethiols analysed in this study, together with linear fit (orange line) used to determine the β and $\log(J_0)$.

6. Computational details

6.1. Transport calculations

All transport properties were calculated using the combined density functional theory (DFT) and non-equilibrium Green's function (NEGF) formalism.[12,13] Geometry optimization of N-Heterocyclic Carbenes (NHC) were performed in gas-phase using the Perdew-Burke-Ernzerhof (PBE) functional[14] with Grimme's dispersion corrections (DFT-D)[15] and Becke-Johnson damping, employing double-ζ polarized (DZP) basis set. Structures were relaxed until the forces on all atoms were below 0.02 eV/Å. Next, Au-nNHC-Au junctions were constructed using a 3×3 FCC Au(111) surface unit cell with n = 1 or 2 NHC molecules to model different

packing densities and to capture π-π interactions in the $n = 2$ case. An Au adatom was modelled at the left electrode to interact with the carbene carbon. The junctions were optimized using the same PBE-D functional, with a DZP basis set for the molecular system and a DZ basis set for Au. 4×4×1 Monkhorst-Pack k-point grid was employed for optimization. All Au atoms except the adatom were fixed at the experimental Au(111) lattice positions (lattice constant = 4.08 Å), and relaxation was performed until forces were below 0.04 eV/Å. The geometries of the optimized junctions are freely available at https://doi.org/10.19061/iochem-bd-9-12. For comparison, alkanethiol junctions were modelled using a 2×2 Au(111) cell with the thiol chemisorbed on one side of the electrode, and optimized at the same theoretical level. To compute the transmission function, additional gold layers were included. A 7×7×1 k-point mesh was used for transport calculation.

Hetero-electrode (Au-nNHC-Al) junctions were modelled by replacing the right Au electrode with Al, maintaining the same geometry. This substitution is justified by similar lattice constants of Au (111) and Al (111), 4.08 Å and 4.05 Å, respectively.[16] Transmission functions were calculated at the same level of theory. Finite bias calculations were performed by applying an incremental bias from 0 to 1 in 0.1 V steps. All DFT calculations were carried out using the SIESTA package,[17] and transport simulations were performed using TranSIESTA module.[12]

6.2. Calculation at wide band limit (WBL)

To investigate the effect of charging on transport properties, spin-polarized geometry optimizations were performed for neutral, cationic, and anionic NHC molecules using the PBE-D functional and DZP basis set. Structures were relaxed until atomic forces were below 0.02 eV/ Å.

For transport calculation under the wide band limit (WBL) approximation, the Hamiltonian (H) and overlap matrix (S) were extracted using the sisl post-processing toolkit.[18] The transmission function $T(E)$ was evaluated using the standard expression:

$$T(E) = Tr\{\Gamma_R G(E) \Gamma_L G(E)^{\dagger}\},$$

where the Green's function $G(E)$ is defined as

$$G(E) = [S \cdot E - H - \Sigma_L - \Sigma_R]^{-1}.$$

In WBL approximation, the self-energies of the electrodes ($\Sigma_{L/R}$) are purely imaginary and energy independent:

$$\Sigma_{L/R} = -\frac{i}{2} \Gamma_{L/R}$$

This simplification neglects the real part of the self-energies, which would otherwise induce energy level shifts due to electrode coupling.

The matrix elements for the self-energies, $\Sigma_{L/R}$ represented by atomic orbitals basis $|\chi_{\upsilon}\rangle$ are

$$(\Sigma_{L/R})_{\upsilon\upsilon} = -\frac{i}{2} (\Gamma_{L/R})_{\upsilon\upsilon},$$

where $(\Gamma_{L/R})_{\upsilon\upsilon} = 1$ eV for orbitals of the carbene carbon at the left side and terminal two H atoms at the right side, respectively. The Fermi energy (E_F) was taken from the DFT calculation in SIESTA, located at the mid-gap between the HOMO and LUMO levels, accounting for electronic temperature and occupancy effects.[13] The script for the WBL transport calculations can be found at https://github.com/SolomonHansen/WBA-Trans.

6.3. Discussion of charge transport calculations

The work function of the electrode is known to have a noticeable influence on transmission.[16] To account for this, we modelled the Au-nNHC-Al junction using the same geometry as in Au-nNHC-Au but replacing the right Au electrode with Al, as the Al electrode has a work function (~4.24 eV) similar to EGaIn (~4.1-4.2 eV).[16] However, while the Al electrode

approximates EGaIn in terms of work function, it does not replicate the interaction between NHCs and Ga_2O_3 surface of the EGaIn electrode. The effect of this heterojunction is shown in **Figure S8**. In all Au-nNHC-Al junctions, the transmission peaks shift such that the unoccupied orbitals move further away from E_F. The hetero-electrode setup enhances conductance for all NHCs except NIM^{Me}. The $T(E_F)$ for all Au-nNHC-Al lie below the alkane reference line except for Au-2NIM^{Me}-Al. (see **Figure S9**).

The theoretical results discussed so far correspond to the zero bias condition. However, since the experimental measurements were performed under a finite bias; specifically measured currents were compared at +0.5V for the series of NHCs (see **Figure 2**); it is important to examine how a positive bias influences transmission characteristics. To analyze the effect of finite positive bias, we computed the transmissions of Au-2NHC-Au systems at 0.5 V and 1.0 V (see **Figure S10**). With increasing positive bias, E_L shifts further away from E_F, thereby reducing $T(E)$ around E_F. The $T(E_F)$ at finite positive bias (0.5 V and 1.0 V) for all systems remain below the alkane reference line in accordance with the experimental results. (see **Figure S11**). Here, it is worth mentioning that at finite bias, the energy window of electron transport broadens. Lowering of $T(E)$ around E_F at finite positive bias may be responsible for the observed insulating behavior. For completeness, we have also checked the impact of negative bias and see that under negative bias LUMO remains closer to the bias window, which leads to lesser suppression of conductance as compared to positive bias. This is also reflected in the I-V curves of all Au-2NHC-Au junctions (**Figure S12**). Here it is important to note that asymmetry in the I-V curves is more pronounced for longer NHCs, whereas for IMMe, junction exhibit nearly symmetric behaviour as its resonance peak lies significantly further from the bias window.

To further understand the molecular contribution on the bias dependent LUMO shift, we have performed additional calculations on isolated NHC molecules under externally applied electric

fields along the molecular axis. We find that the dipole moment (μ), the natural population analysis (NPA) charge on the carbene carbon (Q_c), and the frontier molecular orbital energies (E_H and E_L), as well as the HOMO-LUMO gap (ΔE_{H-L}), all exhibit a clear dependence on both the magnitude and direction of the applied field. (see **Figure S13**) These results demonstrate that the NHC motif possesses intrinsic polarizability, which is also expected to impact the transmission characteristics of the junction. A strong Au-carbene (NHC) interaction may also lead to partial or complete ionization of the NHCs at the gold surface. To explore the impact of NHC ionization, we calculated the transmission of anionic and cationic NHCs at the WBL and compared it with the neutral counterpart (see **Figure S14**). $T(E_F)$ was calculated by averaging the contribution from up- and down-spin channels. The analysis suggests that ionization enhances the transmission values at E_F with minimal impact on the shortest NHC (IM^{Me}) but a significant effect on the longest NHC (NIM^{Me}).

Considering that the adatom formation can depend on the NHC backbone, side groups and other factors, the effect of omitting an adatom on the bonding geometry and transmission was tested. To this end we calculated, and compared, the Au-1NHC-Au transmission considering bonding geometry without an adatom (see **Figure S15**). We find that the overall transmission near E_F increases for bonding geometry without an adatom, which we attribute to the shorter electrode separation. The LUMO peaks become broader, which indicates stronger molecule-electrode coupling. Importantly, the overall shape of the transmission curves and the relative alignment of the LUMO peak remain unchanged. The trend of LUMO peak approaching near to E_F with an increasing length of the NHC backbone is preserved.

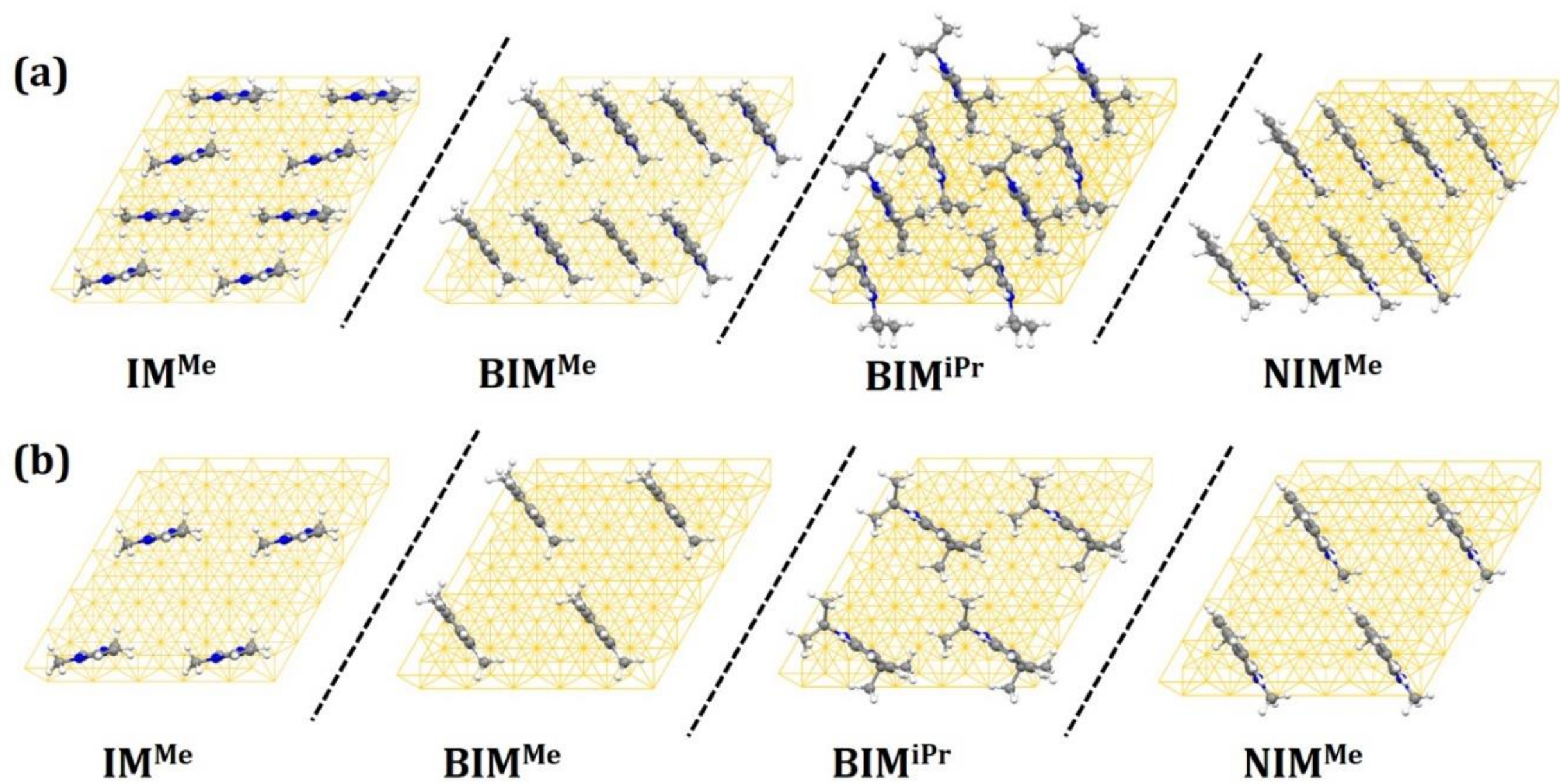

Figure S7. Top-view of the optimized structures of all four NHCs in Au-nNHC-Au ($n = 1,2$) junction, replicated 2 times in two infinite directions. The top and bottom panels display $n = 1$ NHC and $n = 2$ NHCs per 3×3 Au(111) unit cell, respectively. H, C, N, and Au are represented by white, grey, blue and yellow coloured balls respectively.

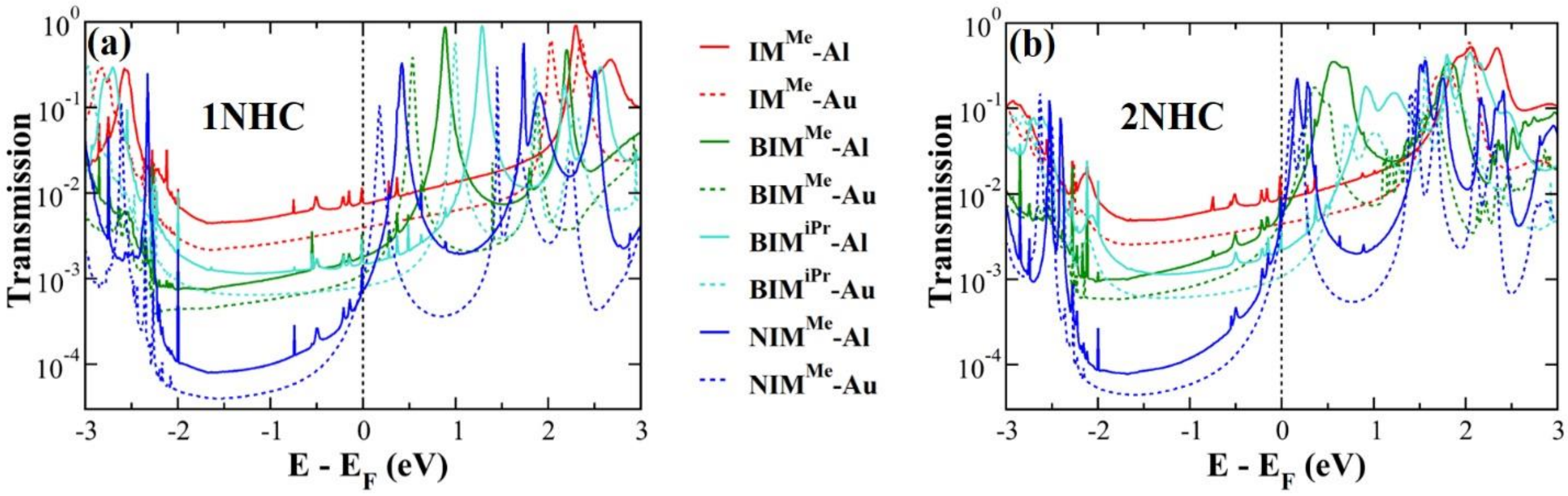

Figure S8. Calculated transmission function per molecule of Au-nNHC-Al/Au ($n = 1$) (a) Au-nNHC-Al/Au ($n = 2$) (b) junction as a function of energy relative to the E_F.

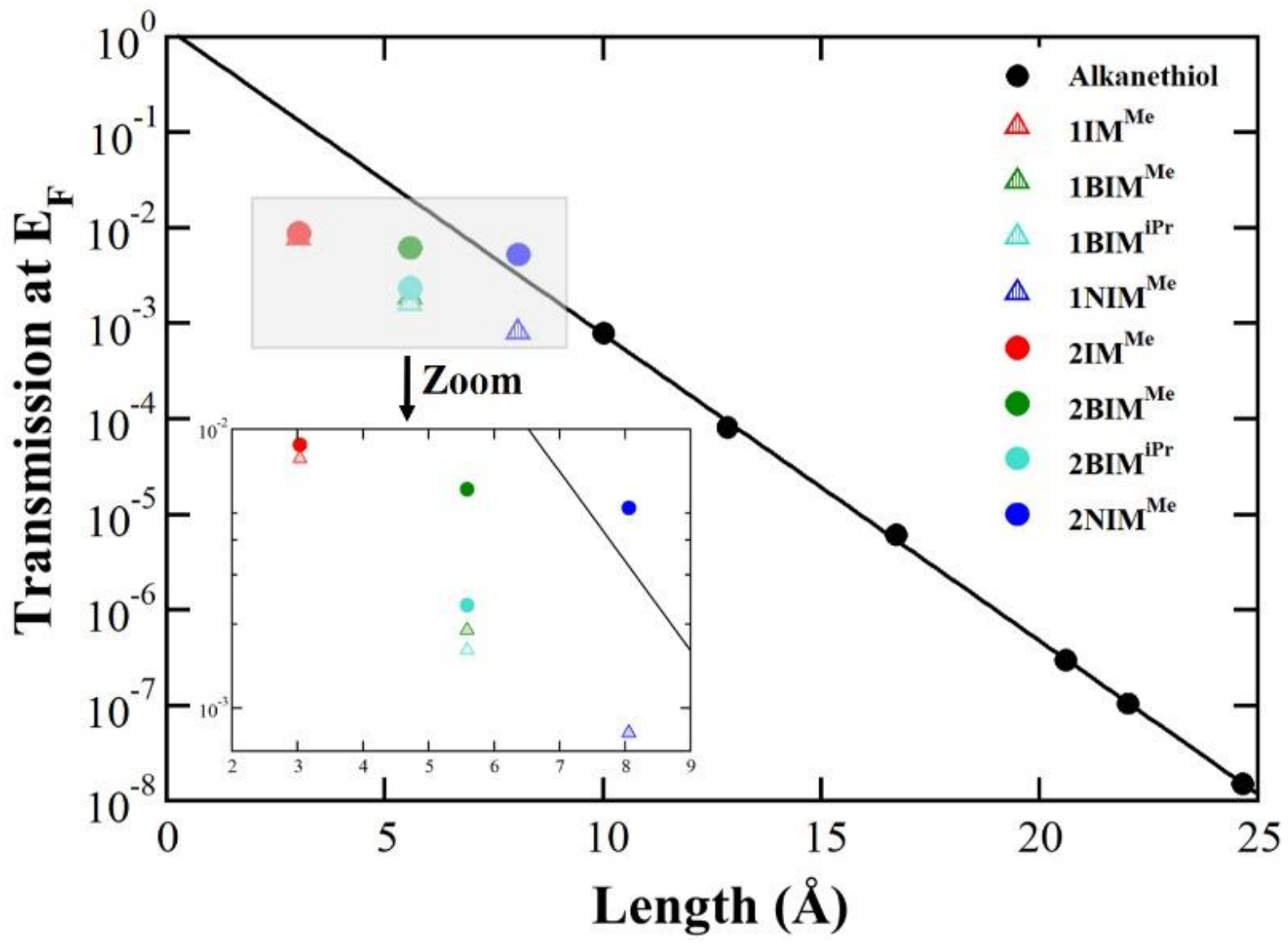

Figure S9. Calculated transmission values per molecule at E_F plotted against molecular length, for Au-nNHC-Al ($n = 1,2$) with alkanethiols as a reference.

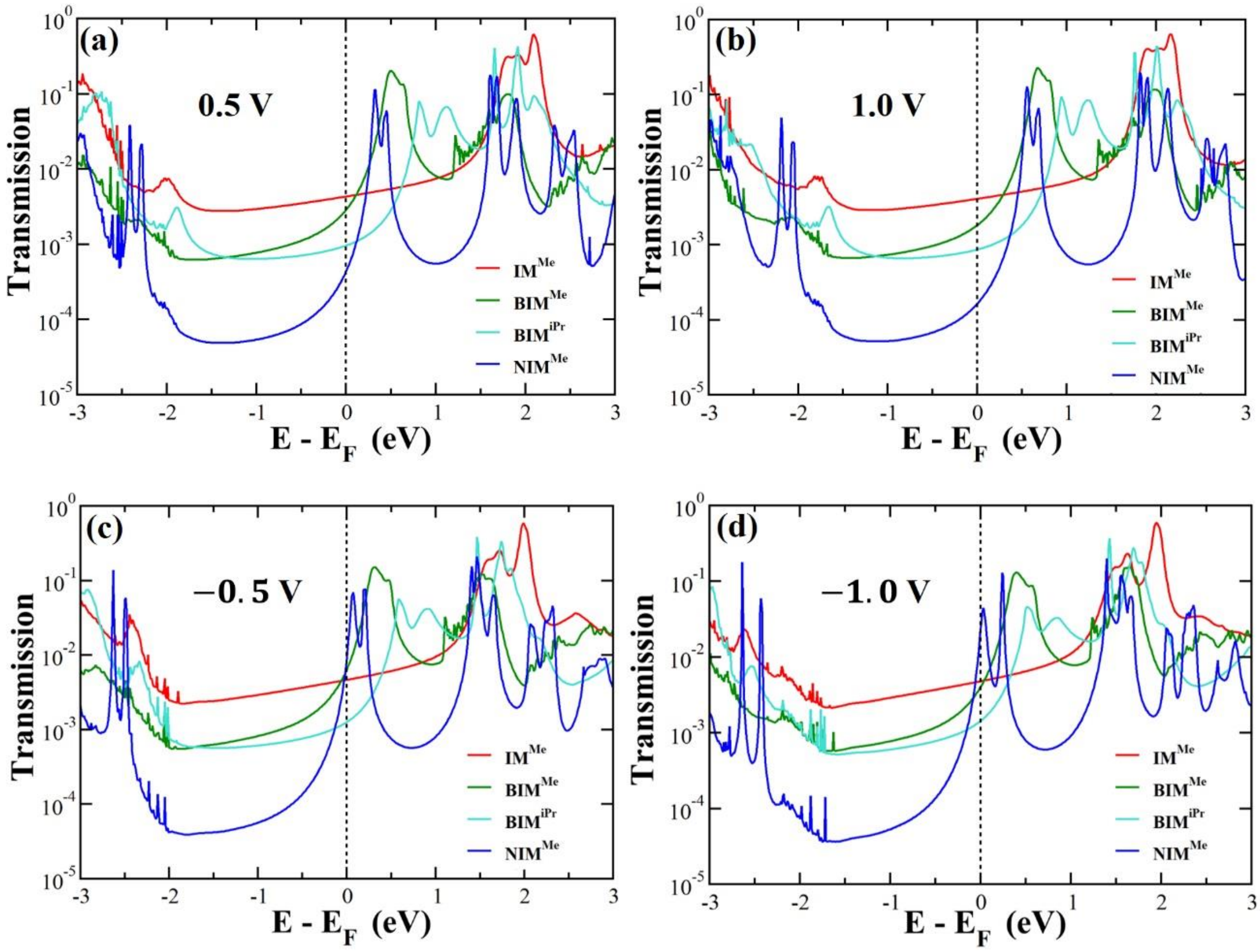

Figure S10. Calculated transmission function per molecule of Au-nNHC-Au ($n = 2$) junction at 0.5 V (a) 1.0 V (b) -0.5 V (c) -1.0 V (d) bias as a function of energy relative to the E_F.

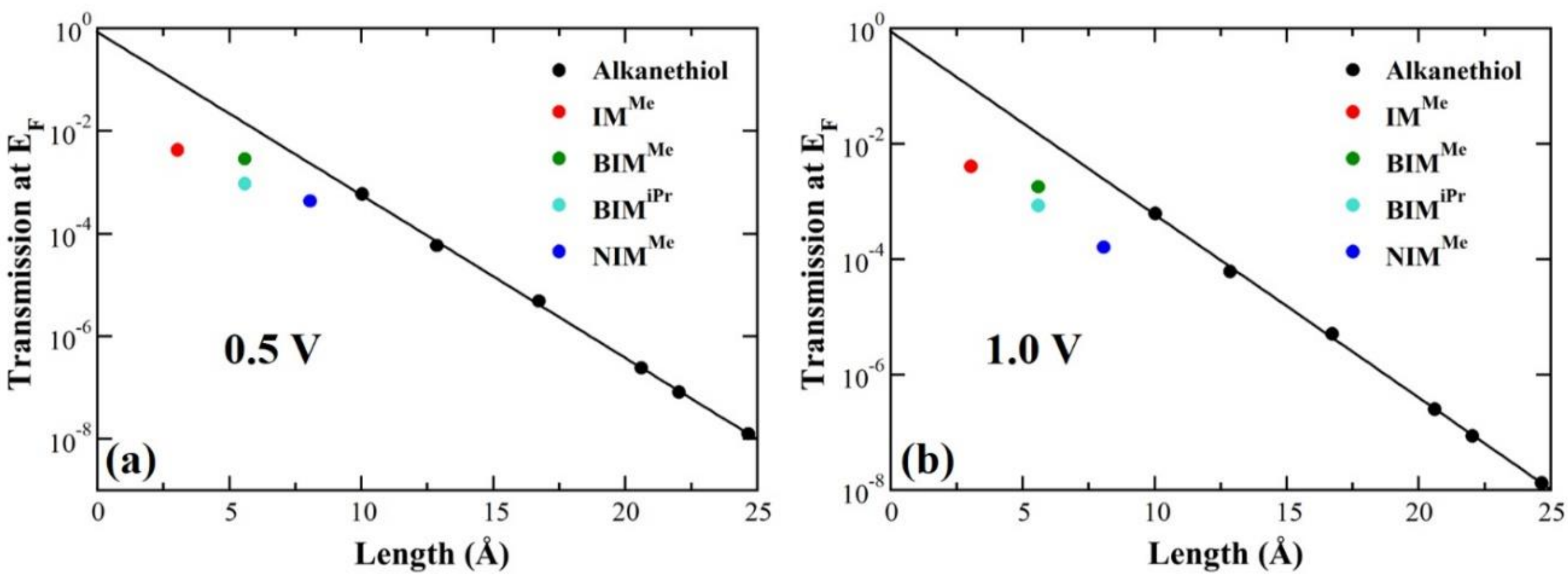

Figure S11. Calculated transmission values per molecule at E_F plotted against molecular length, for Au-nNHC-Au ($n = 2$) at (a) 0.5 V (b) 1.0 V bias with alkanethiols as a reference.

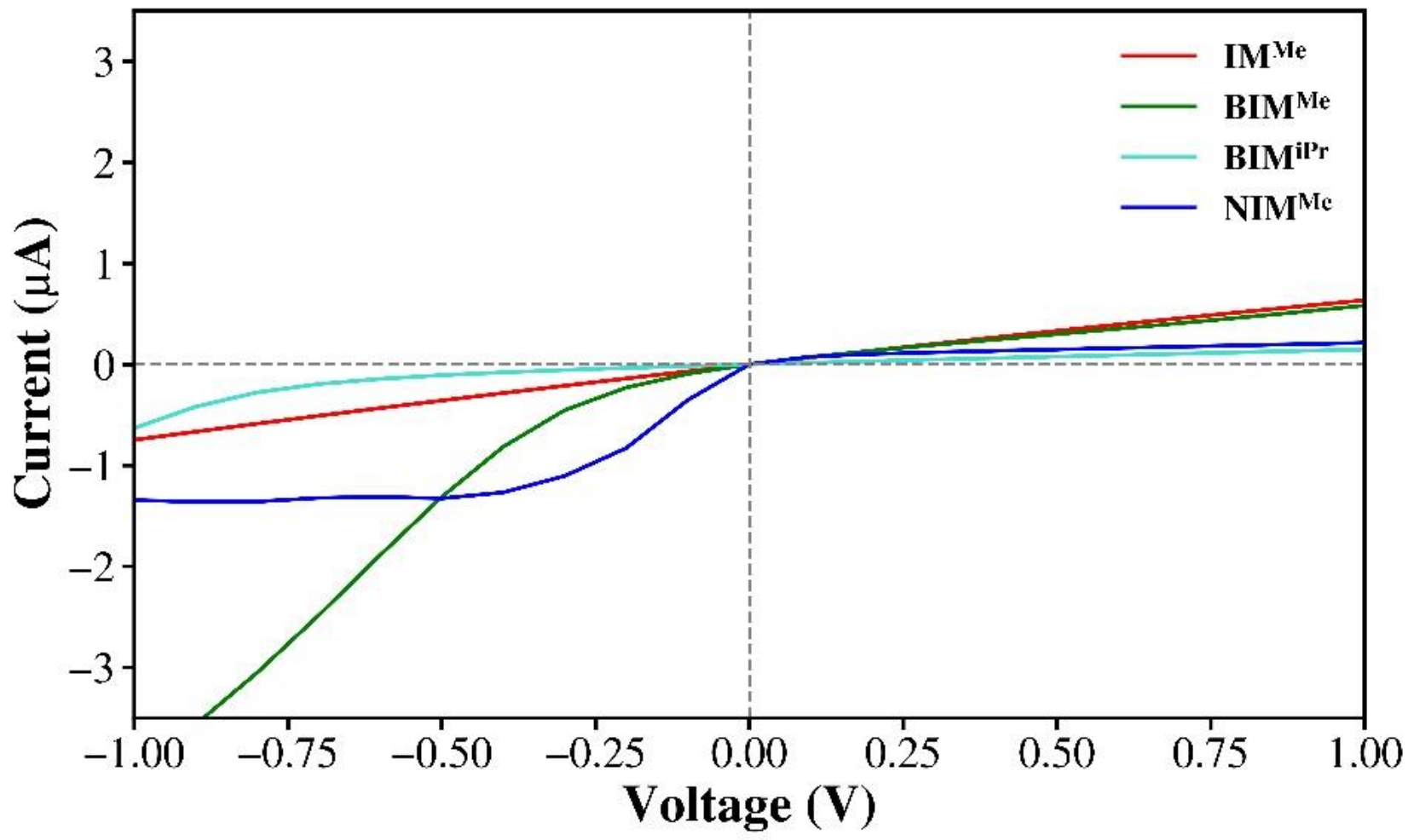

Figure S12: Current-voltage characteristics of all Au-2NHC-Au junctions

	$Z-50$					No Field					$Z+50$				
	μ	E_H	E_L	ΔE_{H-L}	Q_c	μ	E_H	E_L	ΔE_{H-L}	Q_c	μ	E_H	E_L	ΔE_{H-L}	Q_c
**IM^{Me}**	3.3	-8.0	+3.0	11.0	+0.07	2.4	-7.9	+2.8	10.7	+0.10	1.6	-7.7	+2.7	10.4	+0.12
**BIM^{Me}**	3.4	-8.2	+1.4	9.6	+0.12	1.7	-8.1	+1.3	9.4	+0.15	0.1	-7.9	+1.1	9.0	+0.18
**BIM^{iPr}**	3.6	-8.0	+1.5	9.5	+0.12	1.4	-8.0	+1.3	9.3	+0.15	0.8	-7.9	+1.0	8.9	+0.18
**NIM^{Me}**	4.3	-7.4	+0.5	7.9	+0.14	1.3	-7.6	+0.4	8.0	+0.17	1.8	-7.7	+0.2	7.9	+0.21

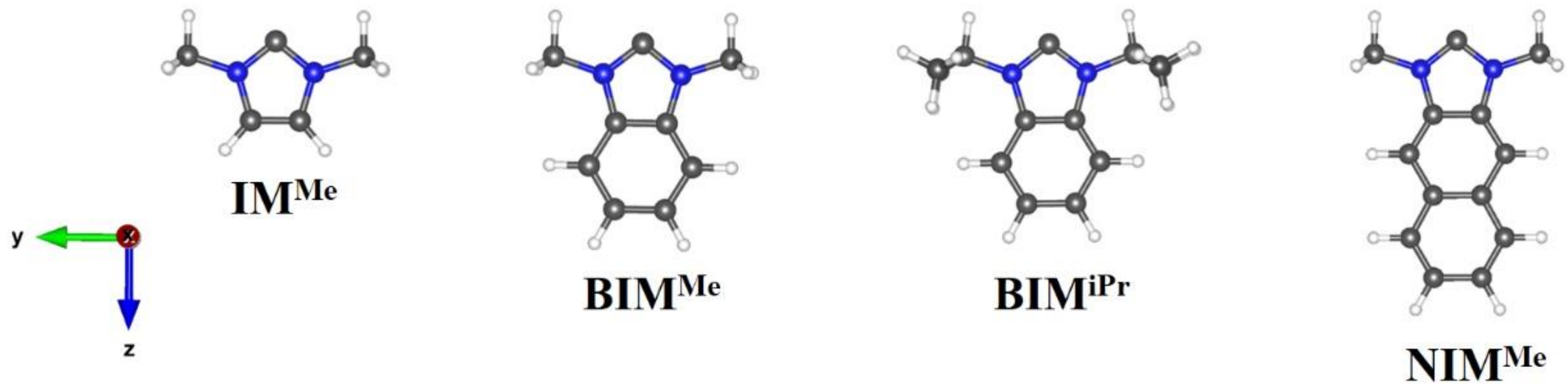

Figure S13: Top panel: Variation of dipole moment (μ) in Debye, HOMO-LUMO energies (E_H, E_L) and gap (ΔE_{H-L}) in eV, charge on carbene carbon (Q_c) by natural population analysis with electric field applied along Z-direction. Z+50 and Z-50 denotes 0.005 a.u. electric dipole field applied on +/- Z direction. Bottom panel: Gas-phase optimized structures of all four NHCs aligned in YZ plane. All these calculations are done at ωB97XD/6-311g(d,p) level of theory in Gaussian16 program.[19]

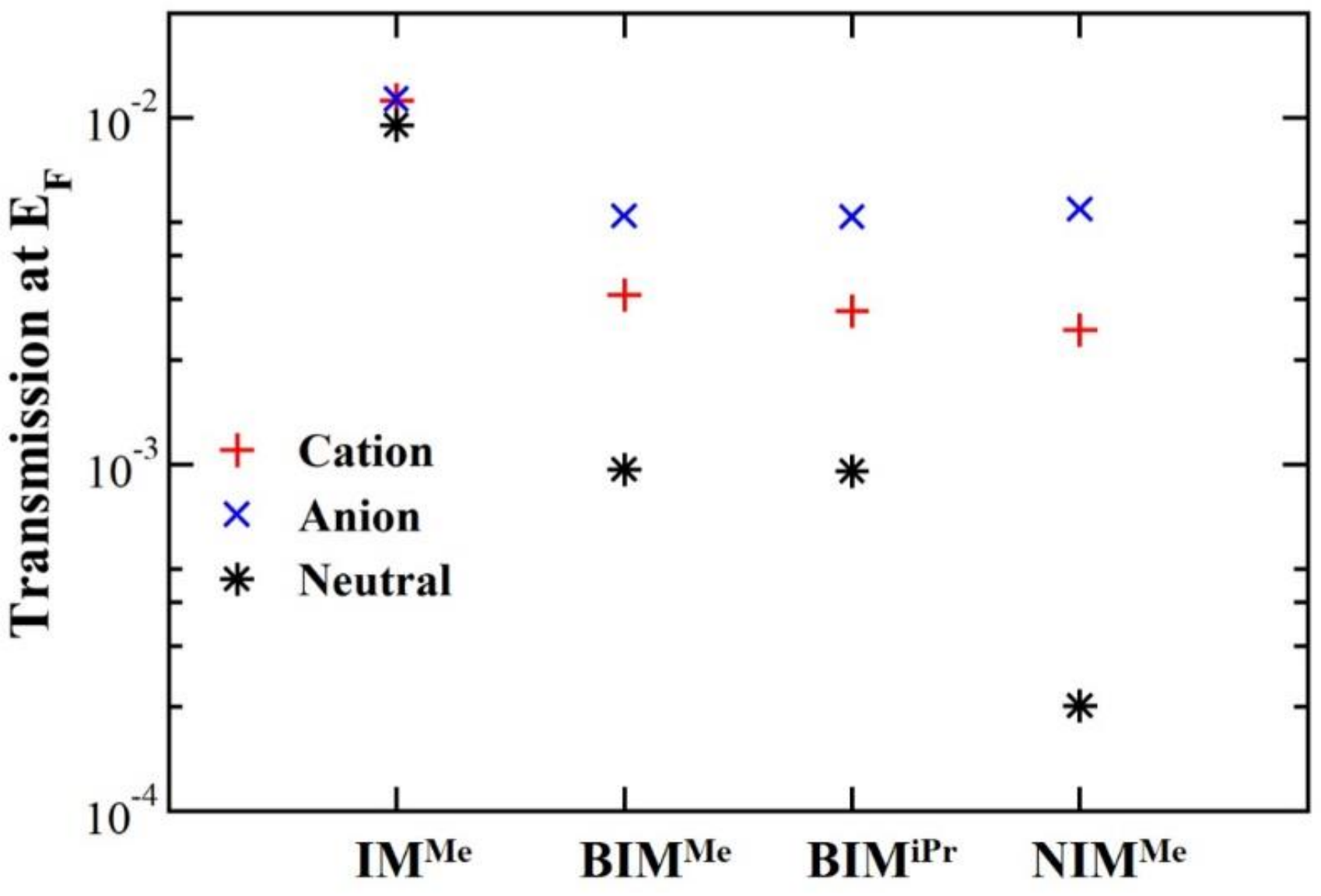

Figure S14. Calculated average transmission values of spin up and down at E_F of all four isolated NHCs in cationic, anionic, and neutral at the wide band limit.

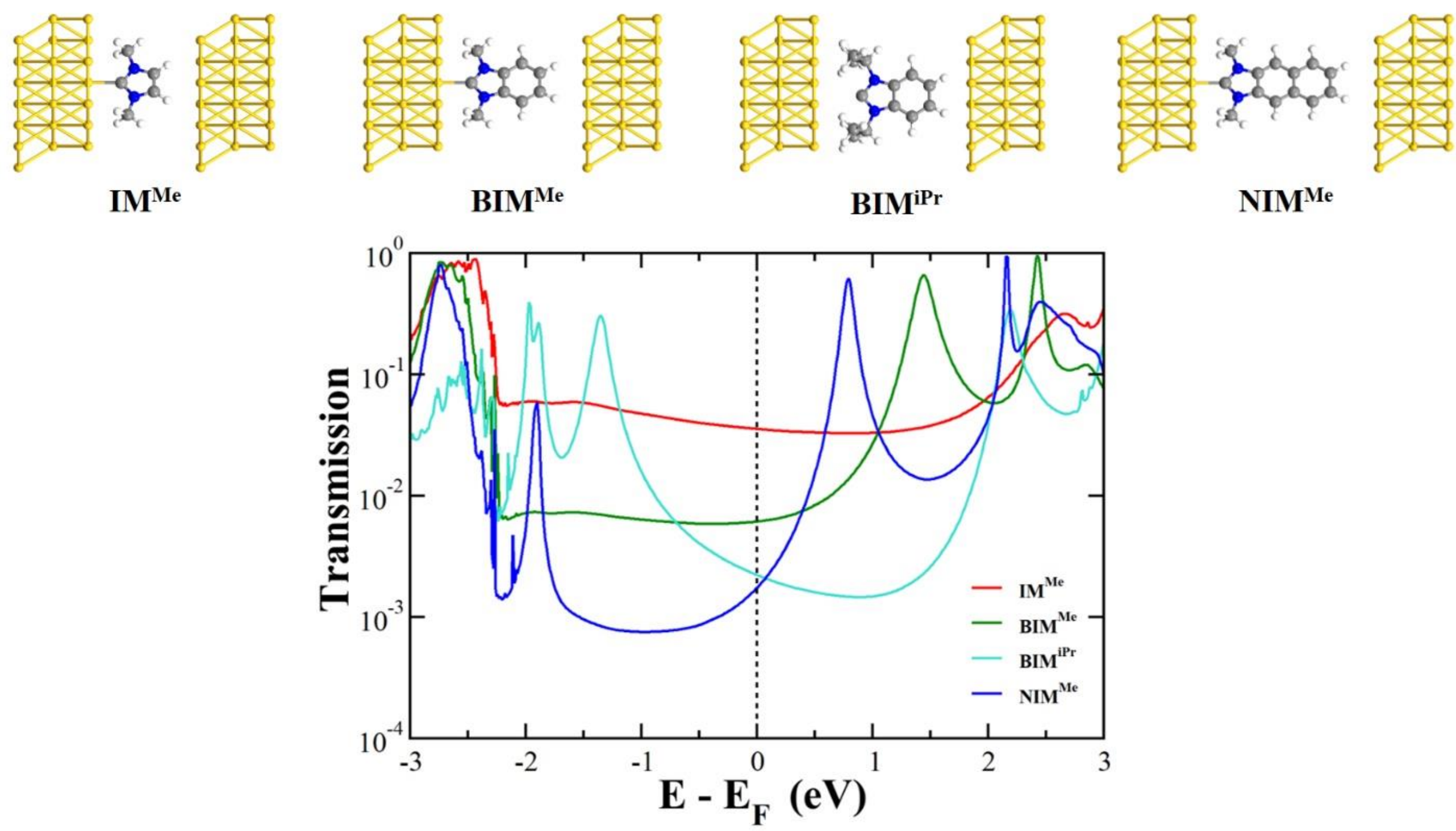

Figure S15. Calculated transmission function of Au-1NHC-Au junction (without an adatom) as a function of energy relative to the E_F.

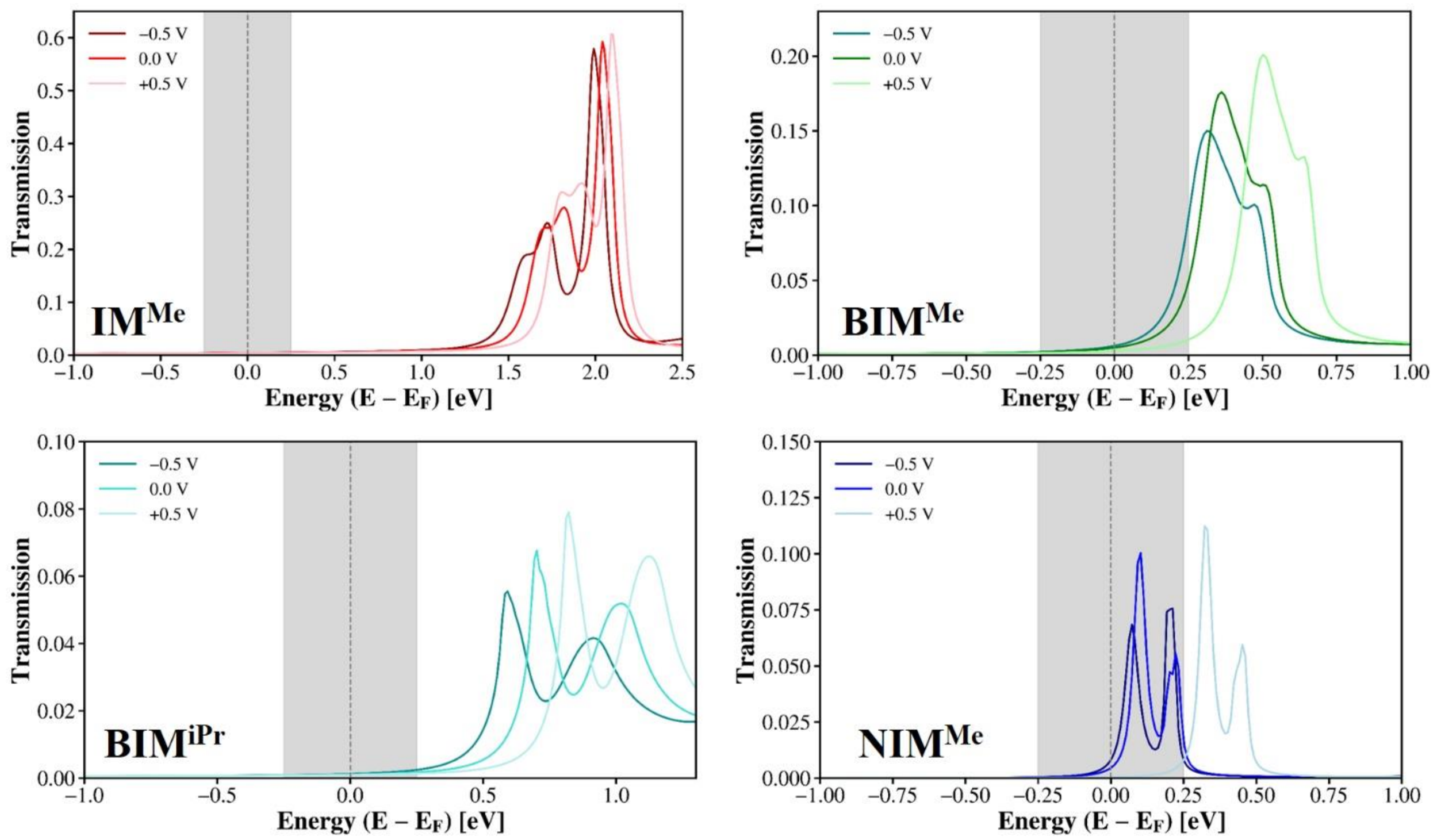

Figure S16: Effective shift of the LUMO resonance relative to the bias window (−0.25 to +0.25 eV) for all four Au-2NHC-Au junctions; the bias window is indicated by the grey shaded region.

Table S1. Calculated frontier molecular orbital energies (in eV) of all four isolated NHCs in gas phase with PBE-D/DZP and ωB97XD/6-311g(d,p) level of theory.

		H-1	H	L	L+1	ΔE_{H-L}
**PBE/DZP**	**IM^{Me}**	-4.70	-3.74	0.61	1.10	4.35
	**BIM^{Me}**	-4.96	-3.99	-0.73	-0.01	3.26
	**BIM^{iPr}**	-4.93	-3.94	-0.73	+0.03	3.21
	**NIM^{Me}**	-4.41	-4.10	-1.48	-0.32	2.62
**ωB97XD/ 6-311g(d,p)**	**IM^{Me}**	-8.26	-7.86	2.81	3.42	10.67
	**BIM^{Me}**	-8.28	-8.14	1.27	2.06	9.41
	**BIM^{iPr}**	-8.22	-8.05	1.26	2.15	9.31
	**NIM^{Me}**	-8.26	-7.58	0.38	1.16	7.96

Table S2. Calculated transmission values per molecule at Fermi energy (E_F) for Au-nNHC-Au/Al ($n = 1,2$) at zero and finite positive bias (V).

	Au-nNHC-Au ($V = 0$)		Au-nNHC-Al ($V = 0$)		Au-2NHC-Au ($V \neq 0$)	
	$n = 1$	$n = 2$	$n = 1$	$n = 2$	$V = 0.5$	$V = 1.0$
**IM^Me**	3.95×10^{-3}	4.46×10^{-3}	7.84×10^{-3}	8.78×10^{-3}	4.28×10^{-3}	4.06×10^{-3}
**BIM^Me**	1.12×10^{-3}	5.02×10^{-3}	1.90×10^{-3}	6.08×10^{-3}	2.83×10^{-3}	1.79×10^{-3}
**BIM^iPr**	8.14×10^{-4}	1.07×10^{-3}	1.61×10^{-3}	2.33×10^{-3}	9.52×10^{-4}	8.55×10^{-4}
**NIM^Me**	9.62×10^{-4}	4.77×10^{-3}	8.13×10^{-4}	5.21×10^{-3}	4.29×10^{-4}	1.61×10^{-4}

7. References

[1] M. Wróbel, D. M. Cegiełka, A. Asyuda, K. Kozieł, M. Zharnikov, P. Cyganik, "N-heterocyclic carbenes – The design concept for densely packed and thermally ultra-stable aromatic self-assembled monolayers" *Nano Today* **2023**, *53*, 102024.

[2] E. A. Weiss, G. K. Kaufman, J. K. Kriebel, Z. Li, R. Schalek, G. M. Whitesides, "Si/SiO2-templated formation of ultraflat metal surfaces on glass, polymer, and solder supports: Their use as substrates for self-assembled monolayers" *Langmuir* **2007**, *23*, 9686–9694.

[3] A. Chandran, N. L. Dominique, G. Kaur, V. Clark, P. Nalaoh, L. C. Ekowo, I. M. Jensen, M. D. Aloisio, C. M. Crudden, N. Arroyo-Currás, D. M. Jenkins, J. P. Camden, "Forming N-heterocyclic carbene monolayers: not all deposition methods are the same" *Nanoscale* **2025**, *17*, 5413–5428.

[4] A. Krzykawska, M. Wróbel, K. Kozieł, P. Cyganik, "N-Heterocyclic Carbenes for the Self-Assembly of Thin and Highly Insulating Monolayers with High Quality and Stability" *ACS Nano* **2020**, *14*, 6043–6057.

[5] C. L. A. Lamont, J. Wilkes, "Attenuation Length of Electrons in Self-Assembled Monolayers of *n* -Alkanethiols on Gold" *Langmuir* **1999**, *15*, 2037–2042.

[6] X. Chen, H. Hu, J. Trasobares, C. A. Nijhuis, "Rectification Ratio and Tunneling Decay Coefficient Depend on the Contact Geometry Revealed by in Situ Imaging of the Formation of EGaIn Junctions" *ACS Appl. Mater. Interfaces* **2019**, *11*, 21018–21029.

[7] W. F. Reus, M. M. Thuo, N. D. Shapiro, C. A. Nijhuis, G. M. Whitesides, **2012**, DOI: 10.1021/nn205089u.

[8] R. C. Chiechi, Available online: https://github.com/rchiechi/gaussfit (accessed on 28 September 2024).

[9] F. C. Simeone, H. J. Yoon, M. M. Thuo, J. R. Barber, B. Smith, G. M. Whitesides, "Defining the Value of Injection Current and Effective Electrical Contact Area for EGaIn-Based Molecular Tunneling Junctions" *J. Am. Chem. Soc.* **2013**, *135*, 18131–18144.

[10] M. Baghbanzadeh, F. C. Simeone, C. M. Bowers, K.-C. Liao, M. Thuo, M. Baghbanzadeh, M. S. Miller, T. B. Carmichael, G. M. Whitesides, "Odd–Even Effects

in Charge Transport across *n* -Alkanethiolate-Based SAMs" *J. Am. Chem. Soc.* **2014**, *136*, 16919–16925.

[11] S. K. Karuppannan, H. Hongting, C. Troadec, A. Vilan, C. A. Nijhuis, "Ultrasmooth and Photoresist-Free Micropore-Based EGaIn Molecular Junctions: Fabrication and How Roughness Determines Voltage Response" *Adv. Funct. Mater.* **2019**, *29*, DOI 10.1002/adfm.201904452.

[12] N. Papior, N. Lorente, T. Frederiksen, A. García, M. Brandbyge, "Improvements on non-equilibrium and transport Green function techniques: The next-generation TRANSIESTA" *Comput. Phys. Commun.* **2017**, *212*, 8–24.

[13] J. M. Soler, E. Artacho, J. D. Gale, A. García, J. Junquera, P. Ordejón, D. Sánchez-Portal, *The SIESTA method for ab initio order-N materials simulation*, **2002**.

[14] J. P. Perdew, K. Burke, M. Ernzerhof, "Generalized Gradient Approximation Made Simple" *Phys. Rev. Lett.* **1996**, *77*, 3865–3868.

[15] S. Grimme, S. Ehrlich, H. Krieg, "A Consistent and Accurate ab initio Parametrization of Density Functional Dispersion Correction (DFT-D) for the 94 Elements H-Pu" *J. Chem. Phys.* **2010**, *132*, 154104.

[16] H. Kang, J. Jang, G. D. Kong, S. Jung, T. Ohto, H. J. Yoon, "Deposition condition impacts charge tunneling and thermoelectric properties of N-heterocyclic carbene monolayers" *J. Mater. Chem. A Mater.* **2023**, *11*, 16233–16242.

[17] A. García, N. Papior, A. Akhtar, E. Artacho, V. Blum, E. Bosoni, P. Brandimarte, M. Brandbyge, J. I. Cerdá, F. Corsetti, R. Cuadrado, V. Dikan, J. Ferrer, J. Gale, P. García-Fernández, V. M. García-Suárez, S. García, G. Huhs, S. Illera, R. Korytár, P. Koval, I. Lebedeva, L. Lin, P. López-Tarifa, S. G. Mayo, S. Mohr, P. Ordejón, A. Postnikov, Y. Pouillon, M. Pruneda, R. Robles, D. Sánchez-Portal, J. M. Soler, R. Ullah, V. W. Yu, J. Junquera, "SIESTA: Recent developments and applications" *J. Chem. Phys.* **2020**, *152*, DOI 10.1063/5.0005077.

[18] N. Papior, P. Febrer Calabozo, "sisl", DOI 10.5281/zenodo.15557193.

[19] M. J. Frisch, G. W. Trucks, H. B. Schlegel, G. E. Scuseria, M. A. Robb, J. R. Cheeseman, G. Scalmani, V. Barone, G. A. Petersson, H. Nakatsuji, X. Li, M. Caricato, A. V. Marenich, J. Bloino, B. G. Janesko, R. Gomperts, B. Mennucci, H. P. Hratchian, J. V. Ortiz, A. F. Izmaylov, J. L. Sonnenberg, D. Williams-Young, F. Ding, F. Lipparini, F. Egidi, J. Goings, B. Peng, A. Petrone, T. Henderson, D. Ranasinghe, V. G. Zakrzewski, J. Gao, N. Rega, G. Zheng, W. Liang, M. Hada, M. Ehara, K. Toyota, R. Fukuda, J. Hasegawa, M. Ishida, T. Nakajima, Y. Honda, O. Kitao, H. Nakai, T. Vreven, K. Throssell, J. A. Montgomery, Jr., J. E. Peralta, F. Ogliaro, M. J. Bearpark, J. J. Heyd, E. N. Brothers, K. N. Kudin, V. N. Staroverov, T. A. Keith, R. Kobayashi, J. Normand, K. Raghavachari, A. P. Rendell, J. C. Burant, S. S. Iyengar, J. Tomasi, M. Cossi, J. M. Millam, M. Klene, C. Adamo, R. Cammi, J. W. Ochterski, R. L. Martin, K. Morokuma, O. Farkas, J. B. Foresman, and D. J. Fox, "Gaussian 16", Gaussian, Inc.: Wallingford, CT, 2016.
